# Shared genetic etiology between Parkinson’s disease and blood levels of specific lipids

**DOI:** 10.1038/s41531-021-00168-9

**Published:** 2021-03-05

**Authors:** Helena Xicoy, Cornelius JHM Klemann, Ward De Witte, Marijn B Martens, Gerard JM Martens, Geert Poelmans

**Affiliations:** 1 Department of Cell Biology, Radboud Institute for Molecular Life Sciences, Radboud University Medical Center, Nijmegen, The Netherlands; 2 Department of Molecular Animal Physiology, Donders Institute for Brain, Cognition and Behaviour, Radboud Institute for Molecular Life Sciences, Radboud University, Nijmegen, The Netherlands; 3 Department of Human Genetics, Radboud University Medical Center, Nijmegen, The Netherlands; 4 NeuroDrug Research, Ltd., Nijmegen, The Netherlands

**Keywords:** Genomics, Parkinson's disease

## Abstract

Parkinson’s disease (PD) is characterized by the degeneration of dopaminergic neurons in the substantia nigra and the formation of Lewy bodies. The mechanisms underlying these molecular and cellular effects are largely unknown. Previously, based on genetic and other data, we built a molecular landscape of PD that highlighted a central role for lipids. To explore which lipid species may be involved in PD pathology, we used published genome-wide association study (GWAS) data to conduct polygenic risk score-based analyses to examine putative genetic sharing between PD and blood levels of 370 lipid species and lipid-related molecules. We found a shared genetic etiology between PD and blood levels of 25 lipids. We then used data from a much-extended GWAS of PD to try and corroborate our findings. Across both analyses, we found genetic overlap between PD and blood levels of eight lipid species, namely two polyunsaturated fatty acids (PUFA 20:3n3-n6 and 20:4n6), four triacylglycerols (TAG 44:1, 46:1, 46:2, and 48:0), phosphatidylcholine aa 32:3 (PC aa 32:3) and sphingomyelin 26:0 (SM 26:0). Analysis of the concordance—the agreement in genetic variant effect directions across two traits—revealed a significant negative concordance between PD and blood levels of the four triacylglycerols and PC aa 32:3 and a positive concordance between PD and blood levels of both PUFA and SM 26:0. Taken together, our analyses imply that genetic variants associated with PD modulate blood levels of a specific set of lipid species supporting a key role of these lipids in PD etiology.

## Introduction

Parkinson’s disease (PD) is the second most common neurodegenerative disease, with a lifetime risk of 2% for men and 1.3% for women^1,2^. PD is characterized by a progressive loss of dopaminergic neurons that project from the substantia nigra (SN) to the striatum, the formation of so-called Lewy bodies (abnormal protein aggregates containing α-synuclein), and microgliosis^3^. The molecular mechanisms underlying these pathological hallmarks have predominantly been studied in familial forms of PD—which account for only 5–10% of the cases—or in animal models of toxin-induced PD (e.g., use of 1-methyl-4-phenyl-1,2,3,6-tetrahydropyridine (MPTP), rotenone, or 6-hydroxydopamine)^4–7^. The etiology and pathophysiology of sporadic PD have not been elucidated, which hampers the development of effective, disease-modifying treatments. To acquire understanding of the mechanisms linked to (sporadic) PD, we previously used the results from genome-wide association studies (GWASs) and other (genetic) data from familial and sporadic PD patients to build a molecular landscape of the disease^8^. This unbiased, hypothesis-generating approach not only confirmed the processes and pathways that have been previously implicated in PD pathology (i.e., oxidative stress, endosomal–lysosomal function, endoplasmic reticulum stress, and a disturbed immune response) but also revealed that lipids play a central role in these processes and hence in PD etiology.

Lipids are mainly known for their role in energy storage^9,10^, but they are also the main constituent of cellular membranes, and part of membrane rafts and anchors as well as signaling and transport molecules^11–15^. According to LIPID MAPS, lipids are classified into eight different classes, namely fatty acyls, glycerolipids, glycerophospholipids, sphingolipids, sterols, prenols, saccharolipids, and polyketides^16^. This classification of lipids is based on their chemical and biochemical properties. In light of the data availability, we focus in this study on the lipids belonging to the first five classes, of which the structural characteristics are shown in Supplementary Fig. 1.

In short, fatty acyls are lipids synthesized by chain elongation of acetyl-CoA and are the building blocks of complex lipids. They include saturated fatty acids (such as palmitic acid), monounsaturated fatty acids (MUFA, such as oleic acid), polyunsaturated fatty acids (PUFAs, such as linoleic acid and docosahexaenoic acid), and fatty acid esters (such as acylcarnitines(AC)). Glycerolipids are composed of mono-, di-, and tri-substituted glycerols, such as monoacylglycerols (MAG), diacylglycerol (DAG), and triacylglycerol (TAG).

Glycerophospholipids (or phospholipids) have a glycerol backbone and a polar headgroup that allows the distinction of several subclasses, including phosphatidylcholine (PC), lysophosphatidylcholine (LPC), and lysophosphatidylethanolamine (LPE). Sphingolipids, such as sphingomyelin (SM), have a sphingoid base backbone synthesized from serine. Lastly, sterols are molecules with a fused four-ring core structure, and they include lipids such as cholesterol and cholesterol esters (CE).

Blood and cellular composition and levels are regulated by multiple factors, such as lipid intake^17–19^, gut microbiota^20^, microRNAs (e.g., miR-33 and miR-122)^21^, and regulatory proteins, e.g., sterol regulatory element-binding proteins, liver X receptors, p53, and AMPK^22,23^. Further, plasma transport of lipids like TAG, phospholipids, cholesterol, and CE occurs in complexes with apolipoproteins, creating lipoproteins, which can be classified into high-density lipoproteins (HDL), intermediate-density lipoproteins (IDL), low-density lipoproteins (VDL), and very-low-density lipoproteins (VLDL)^24^. In addition, the transport of fatty acids occurs in association with other proteins, such as albumin^25^. Therefore, variation in the genes encoding lipid-associated proteins may have a large effect on lipid regulation and disease outcome.

In order to examine the genetic overlap between PD and blood lipid levels, we used polygenic risk score (PRS)-based analysis to determine the extent of shared genetic etiology between PD and the blood levels of 370 lipids and lipid-related molecules, including fatty acyls, glycerolipids, glycerophospholipids, sphingolipids, sterols, and lipoproteins.

## Results

### Shared genetic etiology analyses

**Fig. 1:**
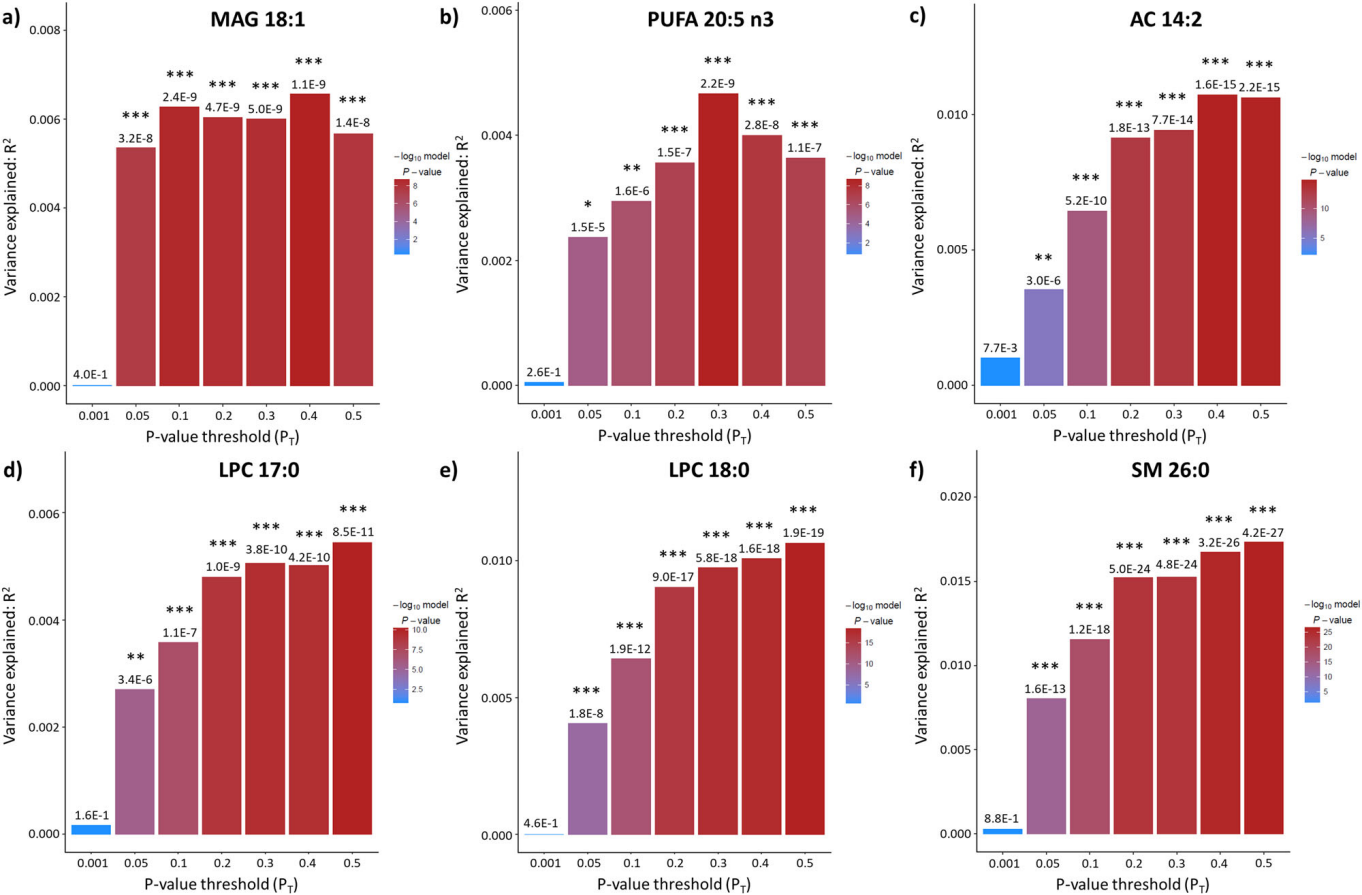
Shared genetic etiology between Parkinson’s disease (PD) and blood levels of MUFA 18:1, PUFA 20:5n3, AC 14:2, LPC 17:0, LPC 18:0, and SM 26:0. Bar plots for shared genetic etiology between PD and the blood levels of monounsaturated fatty acid 18:1 (**a**), polyunsaturated fatty acid 20:5n3 (**b**), acylcarnitine (AC) 14:2 (**c**), lysophosphatidylcholine (LPC) 17:0 (**d**), lysophosphatidylcholine (LPC) 18:0 (**e**), and sphingomyelin (SM) 26:0 (**f**) showing the variance explained (*R*^2^) and the SNP *P* value threshold (*P*_T_). The asterisks above the bars indicate the Bonferroni-corrected significance of the genetic overlap between PD and the blood lipid levels; * denotes *P* < 0.05/2590 tests (7 thresholds × 370 blood lipid levels) = 1.93E-05, ** denotes *P* < 0.01/2590 = 3.86E-06; *** denotes *P* < 0.001/2590 = 3.86E-07.

**Fig. 2:**
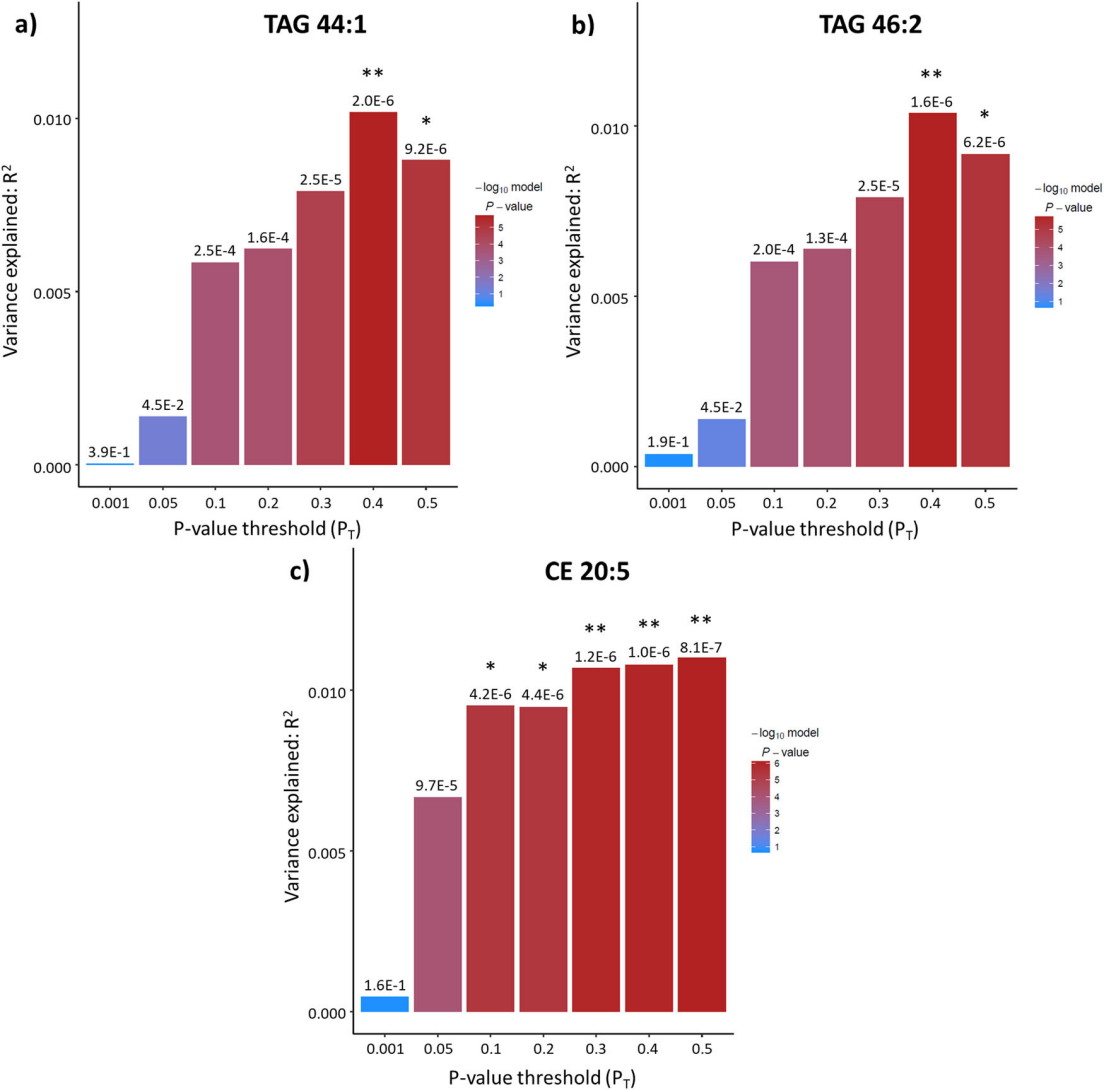
Shared genetic etiology between Parkinson’s disease (PD) and blood levels of TAG 44:1, TAG 46:2, and CE 20:5. Bar plots for shared genetic etiology between PD and the blood levels of triacylglycerol (TAG) 44:1 (**a**), triacylglycerol (TAG) 46:2 (**b**), and cholesterol ester (CE) 20:5 (**c**) showing the variance explained (*R*^2^) and the SNP *P* value threshold (*P*_T_). The asterisks above the bars indicate the Bonferroni-corrected significance of the genetic overlap between PD and the blood lipid levels; * denotes *P* < 0.05/2590 tests (7 thresholds × 370 blood lipid levels)=1.93E-05, ** denotes *P* < 0.01/2590 = 3.86E-06; *** denotes *P* < 0.001/2950 = 3.86E-07.

In this study, we determined the presence and extent of shared genetic etiology between PD and the blood levels of 370 lipids and lipid-related molecules. In phase I, we detected genetic overlap (at least one *P*_T_ showing statistical significance after Bonferroni correction, i.e., *P* < 1.93E-05) between PD and the plasma levels of 25 lipids (Table 1). A complete overview of the results of all PRS-based analyses is shown in Supplementary Data 1. Of note, we found prominent genetic sharing between PD and the blood levels of six specific lipids (MAG 18:1, PUFA 20:5n3, AC 14:2, LPC 17:0, LPC 18:0, and SM 26:0) as each of these lipids showed significance—after Bonferroni correction—at all *P*_T_s, except for the lowest one (*P*_T_ = 0.001) (Fig. 1). Further, genetic variants associated with PD explain at least 1% of the variation in blood levels of six lipids, i.e., the aforementioned AC 14:2, LPC 18:0, and SM 26:0, as well as TAG 44:1, TAG 46:2, and CE 20:5 (Figs. 1 and 2).

**Table 1:** Summary of the results of the phase I PRS-based analyses of the genetic sharing between PD and the blood levels of 370 lipids and lipid-related molecules.

Lipid class	Lipid sub-class	Total number of lipids	Number of lipids with *P* < 1.93E-05	Number of lipids with *R*^2^ > 1%
Fatty acyls	Fatty acids	60	4	0
	AC	23	4	1
Glycerolipids	Metabolism	2	0	0
	MAG	4	1	0
	DAG	4	0	0
	TAG	47	4	2
Glycerophospholipids	PC aa	37	1	0
	PC ae	36	3	0
	LPC	14	3	1
	LPE	6	0	0
	LPI	3	0	0
	Inositol metabolism	3	0	0
Sphingolipids	SM	10	2	1
Sterols	CE	11	1	1
	Cholesterol	3	0	0
	Other	12	1	0
Lipoproteins	HDL	24	1	0
	IDL	6	0	0
	LDL	16	0	0
	VLDL	32	0	0
	Apolipoproteins	2	0	0
Others	Bile acid metabolism	11	0	0
	Others	4	0	0

*AC* acylcarnitine, *MAG* monoacylglycerol, *DAG* diacylglycerol, *TAG* triacylglycerol, *PC* phosphatidylcholine, *PD* Parkinson’s disease, *aa* diacyl, *ae* acyl-alkyl, *LPC* lysophosphatidylcholine, *LPE* lysophosphatidylethanolamine, *LPI* lysophosphatidylinositol, *SM* sphingomyelin, *CE* cholesterol ester, *HDL* high-density lipoprotein, *IDL* intermediate-density lipoprotein, *LDL* low-density lipoprotein, *VDLD* very-low-density lipoprotein.

Listed are the total number of lipid species examined per lipid class, the number of lipids that show Bonferroni-corrected significant genetic sharing (*P* < 1.93E-05) with PD for at least one SNP *P* value threshold (*P*_T_), and the number of lipids for which genetic variants associated with PD explain >1% of the variance (*R*^2^) in blood levels. In total, we found 25 lipids displaying significant genetic sharing with PD.

We then aimed to corroborate our results using a larger PD GWAS study as “base sample”. In phase II, we confirmed a significant shared genetic etiology—after Bonferroni correction, i.e., *P* < 0.05/175 tests (7 thresholds × 25 blood lipid levels) = 2.86E-04)—between PD and the blood levels of eight out of the 25 lipid species that we identified in phase I: PUFA 20:3n3 or n6, PUFA 20:4n6, TAG 44:1, TAG 46:1, TAG 46:2, TAG 48:0, PC aa 32:3, and SM 26:0 (Table 2).

**Table 2:** Corroboration of the genetic overlap between PD and the blood levels of 25 lipids displaying significant genetic sharing with PD in phase I.

	Phase I			Phase II		
	*P*_T_	*P* value	*R*^2^	*P*_T_	*P* value	*R*^2^
18:4n3	0.3	**1.34E-07**	3.60E-03	0.4	4.82E-02	3.77E-04
20:3n3 or n6	0.2	**4.12E-07**	3.30E-03	0.5	**3.38E-05**	2.16E-03
20:4n6	0.3	**4.51E-06**	2.67E-03	0.5	**1.33E-06**	2.99E-03
20:5n3	0.3	**2.19E-09**	4.66E-03	0.5	1.17E-03	1.26E-03
AC 2:0	0.2	**6.73E-06**	2.53E-03	0.5	1.70E-02	6.01E-04
AC 8:1	0.2	**1.14E-05**	2.40E-03	0.5	1.25E-02	6.72E-04
AC 14:2	0.4	**1.61E-15**	1.07E-02	0.1	3.20E-03	1.29E-03
AC 18:0	0.4	**1.11E-06**	3.30E-03	0.1	3.04E-02	5.20E-04
MAG 18:1	0.4	**1.08E-09**	6.57E-03	0.1	4.78E-04	2.01E-03
TAG 44:1	0.4	**2.01E-06**	1.02E-02	0.4	**1.02E-05**	1.01E-02
TAG 46:1	0.4	**1.37E-05**	8.43E-03	0.3	**1.18E-05**	9.90E-03
TAG 46:2	0.4	**1.62E-06**	1.04E-02	0.3	**1.03E-05**	1.00E-02
TAG 48:0	0.4	**1.91E-05**	8.13E-03	0.4	**1.42E-06**	1.21E-02
PC aa 32:3	0.4	**1.85E-05**	2.27E-03	0.5	**1.23E-04**	1.80E-03
PC ae 32:2	0.4	**1.52E-06**	2.91E-03	0.05	2.58E-03	1.05E-03
PC ae 38:1	0.5	**5.14E-06**	2.97E-03	0.3	2.19E-03	1.24E-03
PC ae 44:6	0.4	**8.19E-06**	2.48E-03	0.05	2.79E-02	4.90E-04
LPC 16:0	0.3	**8.82E-07**	3.05E-03	0.001	3.59E-03	9.67E-04
LPC 17:0	0.5	**8.52E-11**	5.44E-03	0.3	9.83E-04	1.28E-03
LPC 18:0	0.5	**1.95E-19**	1.06E-02	0.001	3.39E-03	9.80E-04
SM 26:0	0.5	**4.21E-27**	1.73E-02	0.3	**3.00E-05**	2.45E-03
SM 26:1	0.4	**5.32E-06**	2.59E-03	0.001	6.22E-04	1.39E-03
CE 20:5	0.5	**8.10E-07**	1.10E-02	0.5	1.33E-02	2.73E-03
4-androsten-3beta,17beta-diol disulfate 1	0.5	**3.75E-07**	3.32E-03	0.3	1.12E-03	1.27E-03
HDL-C	0.2	**1.02E-05**	0.000841378	0.05	1.09E-01	7.01E-05

*AC* acylcarnitine, *MAG* monoacylglycerol, *TAG* triacylglycerol, *PC* phosphatidylcholine, *PD* Parkinson’s disease, *aa* diacyl, *ae* acyl-alkyl, *LPC* lysophosphatidylcholine, *SM* sphingomyelin, *CE* cholesterol ester, *HDL* high-density lipoprotein.

Comparison of the most significant *P* value threshold (*P*_T_), *P* value, and explained variance (*R*^2^) for the results obtained in phases I and II. Significant results after Bonferroni correction (phase I: *P* < 1.93E-05; phase II: *P* < 2.86E-04), are highlighted in bold. In total, eight lipids show significant genetic sharing with PD in both phase I and II.

Further, SECA analyses yielded significant evidence—after Bonferroni correction—of genetic pleiotropy (i.e., the same genetic variants affecting two traits) between PD and the blood levels of the eight lipids that were corroborated in phase II.

In addition, in both phase I and II, we found a significant negative genetic concordance between PD and the blood levels of TAG 44:1, TAG 46:1, TAG 46:2, TAG 48:0, and PC aa 32:3, which implies that genetic variants associated with PD contribute to decreased blood levels of these lipids. Conversely, we found a positive concordance between PD and the blood levels of PUFA 20:3n3 or n6, PUFA 20:4n6, and SM 26:0 (Table 3).

**Table 3:** Comparison of the pleiotropy and concordance results generated using SNP effect concordance analysis (SECA) for the eight lipids for which we found significant genetic sharing in both phases I and II.

	Phase I			Phase II		
	*P* value pleiotropy	*P* value concordance	Direction	*P* value pleiotropy	*P* value concordance	Direction
20:3n3 or n6	1	<0.001	+	<0.001	<0.001	+
20:4n6	<0.001	<0.001	+	<0.001	<0.001	+
TAG 44:1	<0.001	<0.001	−	<0.001	<0.001	−
TAG 46:1	<0.001	<0.001	−	<0.001	<0.001	−
TAG 46:2	<0.001	<0.001	−	<0.001	<0.001	−
TAG 48:0	<0.001	<0.001	−	<0.001	<0.001	−
PC aa 32:3	<0.001	<0.001	−	<0.001	<0.001	−
SM 26:0	<0.001	<0.001	+	<0.001	<0.001	+

*TAG* triacylglycerol, *PC* phosphatidylcholine, *aa* diacyl, *SM* sphingomyelin, *CE* cholesterol ester.

The *P* values for genetic pleiotropy (same genetic variants affecting two traits) and concordance (agreement in genetic variant effect directions across two traits) are shown. In addition, for the concordances, the direction of the relationship is indicated by “+” (positive concordance) or “−” (negative concordance). All results except one (pleiotropy between PD and blood levels of 20:3n3 or n6 in phase I) reach Bonferroni-corrected significance (i.e., *P* < 0.05/32 tests (eight tests for pleiotropy and eight tests for concordance in both phase I and II) = 1.56E-03).

## Discussion

Our PRS-based analyses using GWAS data of PD and the blood levels of 370 different lipids yielded a strong genetic link between PD and the blood levels of eight specific lipid species. More specifically, we determined genetic sharing and a positive genetic concordance between PD and the blood levels of two PUFA, namely PUFA 20:3n3 or n6 (also known as eicosatrienoic acid or dihomo-gamma-linoleic acid) and PUFA 20:4n6 (also known as arachidonic acid, AA). Increased levels of AA and dihomo-gamma-linoleic acid have been detected in the cerebrospinal fluid (CSF) of PD patients^26^. Furthermore, PD is associated with an increased intake of AA^27^, although not consistently^28^, and AA is not only linked to increased oxidative stress and neuroinflammation^29,30^ but it also induces α-synuclein aggregation^31^, which are three processes that have been implicated in PD etiology.

We further observed a negative concordance between PD and the blood levels of TAG 44:1, TAG 46:1, TAG 46:2, and TAG 48:0. Although there is no information regarding PD concerning these four specific TAG species, decreased blood levels of TAGs have been repeatedly observed in PD patients compared to controls^32–37^, high blood levels of TAGs have been reported as a protective factor for PD^38,39^, and A53T α-synuclein overexpression leads to decreased serum TAGs levels in animal models^40^. In addition, since TAG is positively correlated with body mass index (BMI)^41^, our results could indicate that PD patients may be genetically predisposed to a lower BMI. This agrees with a previous study that used GWAS data of PD and BMI and, applying Mendelian randomization, found that a higher BMI leads to a lower risk of PD^42^. Similarly, our results are in agreement with a study that used the same methodology but analyzed >5000 risk factors/phenotypic traits and found an inverse relationship between PD risk and adiposity^43^.

However, although we found overlap between PD and blood levels of specific TAGs, we did not observe genetic sharing between PD and total TAG blood levels. This could be partially due to the fact that the blood levels of TAGs are modulated by environmental factors^44–46^, such as diet and microbiome composition which have both been found to differ between PD patients and controls^47,48^.

In addition, we found a negative genetic concordance between PD and the blood levels of PC aa 32:3. PC has an anti-inflammatory role^49^ and it is the most abundant glycerophospholipid in eukaryotic membranes where it is involved in lipid homeostasis^50^. Blood levels of PC aa 32:3 have not been studied in PD but decreased levels of other PC species have been observed in plasma from PD patients^51^. This is in keeping with our finding that genetic variants associated with PD contribute to decreased levels of a specific PC species.

The strongest evidence of genetic sharing that we found was between PD and blood levels of SM 26:0. Furthermore, we found a positive concordance between PD and SM 26:0 blood levels, implying that genetic variants associated with PD contribute to increased levels of this lipid. SM is one of the constituents of the cellular membrane, and it is a source of bioactive lipids that play a role in processes such as autophagy^52^ and cell death^53^. Although it is not known what the blood levels of SM 26:0 in PD are, mutations in *SMPD1*, a gene encoding a sphingomyelin phosphodiesterase, which results in SM accumulation, are a risk factor for PD^54–56^. In addition, increased plasma levels of SM 26:0 have been described in the neurodegenerative disease X-linked adrenoleukodystrophy^57^. Interestingly, several molecular links between PD and X-linked adrenoleukodystrophy have been identified, including α-synuclein accumulation and oxidative stress^58,59^. Hence, elucidation of the physiological and pathological roles of SM 26:0 may contribute to the understanding of the molecular mechanisms underlying multiple neurodegenerative diseases.

Given the above, the genetic overlap between PD and the blood levels of specific lipids can be exploited for the identification of novel diagnostic biomarkers and for the elucidation of the molecular mechanisms underlying PD.

However, the current knowledge on the role of lipids in PD is fragmented^60^, which hinders drawing firm conclusions about the possible links between the disease and blood levels of the eight lipid species for which we found genetic sharing. Moreover, it should be noted that the blood lipid analysis did not make a distinction between lipid isobars (molecules with the same nominal mass) and lipid isomers (molecules with the same molecular formula, but a different chemical structure). For example, TAG 44:1 may correspond to 16 different species, such as TAG 12:0/12:0/20.1, TAG 14:0/14:0/16:1, or TAG 12:0/16:0/16:1. Therefore, the annotated species PUFA 20:3n3 or n6, PUFA 20:4n6, TAG 44:1, TAG 46:1, TAG 46:2, TAG 48:0, PC aa 32:3, and SM 26:0 may correspond to various isobars and isomers, and their exact identity of the species associated with PD thus remains to be determined. In addition, the lack of publicly available data regarding multiple lipid subclasses, such as ceramide-derived lipids and cardiolipin, prevented their inclusion in this study. It should also be noted that the authors cannot be certain that the publicly available data that were used in this study (i.e., the GWASs of blood lipid levels) do not have any overlap with the PD GWAS of phase II, which includes data from participants of the 23andMe consortium. The existence of this overlap could bias the obtained results.

The molecular mechanisms underlying PD have been mainly studied from a genetic, transcriptomic, and/or proteomic perspective, but little is known about the role of the metabolome, and in particular lipids, while our molecular PD landscape indicated a crucial role for lipids in the development of this neurodegenerative disease^8^. In this study, we found genetic sharing between PD risk and blood levels of eight lipids. In future studies, these lipids—including their isobars and isomers—should be further explored before they can possibly be used for the development of, e.g., lipid-directed dietary interventions or lipid-modifying drugs as treatment options to slow or perhaps even stop disease progression.

## Methods

### Shared genetic etiology analyses

In phase I, we used PRSice^61^ to determine the level of shared genetic etiology between PD and the blood levels of 370 different lipids and lipid-related molecules in the population.

As “base sample” for the polygenic risk score (PRS)-based analyses in PRSice (version 1.25), we used summary statistics data for 9581 PD cases and 33,245 matched controls from the PD GWAS reported by Nalls et al.^62^. These data were provided by the University of Tübingen, Germany, and contained data from all participants in phase I of the GWAS, except the participants of the 23andMe consortium. As “target samples” for the PRS-based analyses, we used summary statistics from the GWASs of 370 unique, different blood lipid levels, i.e., GWASs of 74 blood lipid levels from up to 2076 participants from Rhee et al.^63^, GWASs of 89 blood lipid levels from up to 7824 participants from Shin et al.^64^, GWASs of 106 blood lipid levels from up to 7478 participants from Draisma et al.^65^, and GWASs of 101 blood lipid levels from up to 24,925 participants from Kettunen et al.^66^. Before calculating shared genetic etiology, PRSice performed clumping using PLINK (version 1.90)^67^ to select independent index SNPs for each linkage disequilibrium (LD) block in the genome. Subsequently, we used the same approach that we, e.g., applied previously to determine the extent of genetic sharing between autism and autistic traits in the general population^68^. First, based on the significance level of the SNPs in the base sample, the index SNPs were selected and form clumps of all other SNPs that are within 500 kb and are in LD (*r*^2^ > 0.25)^68^. Second, based on the clumped summary statistics of the PD GWAS, PRSice then generated polygenic risk scores that are the sum of genome-wide SNPs associated with PD weighted by their effect sizes estimated from the PD GWAS, from which only the SNPs exceeding seven broad *P* value thresholds (*P*_T_s) were included. The seven thresholds that were used are 0.001, 0.05, 0.1, 0.2, 0.3, 0.4, and 0.5^61^. Subsequently, PRSice calculated *P* values of shared genetic etiology—i.e., the extent to which the combined SNPs from each of the seven *P*_T_-linked polygenic risk scores for PD predict each of the target phenotypes (370 blood lipid levels)—between PD on one hand and each of the 370 blood lipid levels on the other hand. *P* values were considered significant if they exceeded the Bonferroni-corrected threshold accounting for the number of phenotypes tested (*P* < 0.05/2590 tests (7 thresholds × 370 blood lipid levels) = 1.93E-05). Subsequently and using the methodology described above, we tried to corroborate the significant findings from the phase I—i.e., the blood lipid levels for which we found Bonferroni-corrected significant *P* values of shared genetic etiology with PD—through conducting PRS-based analyses with summary statistics from a much larger GWAS of PD as “base sample” and summary statistics from the GWASs of the significant blood lipid levels as “target samples”. For phase II, we used data from the largest GWAS of PD reported thus far that contains all participants (PD cases and controls) in phases I and II from the 2014 GWAS by Nalls et al.^62^ (see above), including the 23andMe participants), as well as a large number of so-called “PD proxy-cases”—defined as those with a first degree relative with PD but no ICD-10 diagnosis or self-report of PD—and multiple new case–control samples. This resulted in GWAS data for a total of 37,688 PD cases, 18,618 PD proxy-cases, and 1,417,791 controls that were provided by the 23andMe consortium^69^.

### SNP effect concordance analyses

We then performed SNP Effect Concordance analysis (SECA) for the corroborated findings from the PRS-based analyses. In SECA (http://neurogenetics.qimrberghofer.edu.au/SECA; see ref. ^70^ for more details), association results rather than individual genotyped data are analyzed to test for genetic pleiotropy (the same SNPs affecting both traits) and concordance (the agreement in SNP effect directions across both traits) between two genetically determined traits.

We used SECA to calculate empirical *P* values for pleiotropy and concordance between all blood lipid levels that emerged from the PRS-based analyses as having a significant shared genetic etiology with PD in both phases I and II. SECA *P* values lower than the Bonferroni-corrected threshold accounting for the number of tests that we performed were considered significant.

### Reporting summary

Further information on experimental design is available in the Nature Research Reporting Summary linked to this paper.

## Supplementary information

Supplementary Information

Reporting summary

Supplementary data 1

## Data Availability

The data that support the findings of this study are available from the corresponding author upon reasonable request.

## Code availability

The code and scripts are available from the corresponding author upon reasonable request.
