# Supplementary material for: Shared genetic etiology between Parkinson’s disease and blood levels of specific lipids
Source: NPJ Parkinsons Dis. 2021 Mar 5;7:23. doi: 10.1038/s41531-021-00168-9 (PMC7935855; doi:10.1038/s41531-021-00168-9)
Supplement: Supplementary file 1 — Supplementary Information [file 41531_2021_168_MOESM1_ESM.pdf]

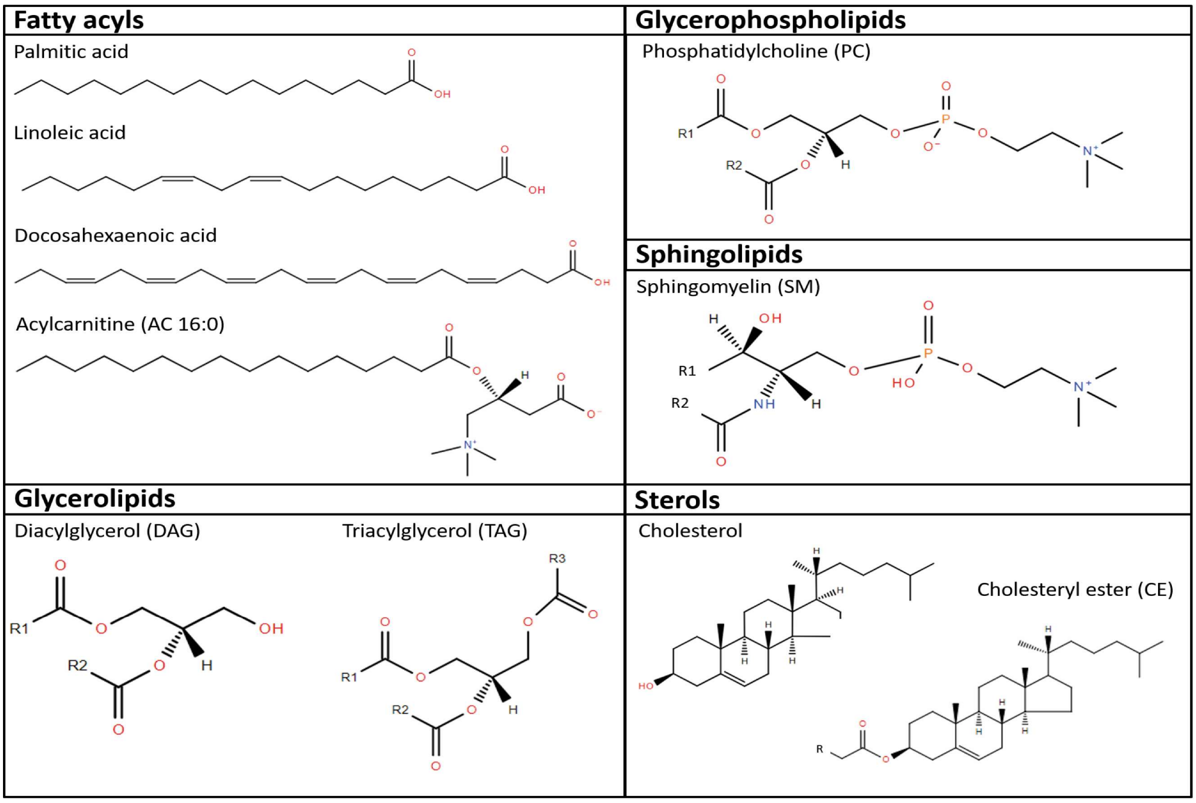

Supplementary figure 1. Chemical structure of relevant lipid classes. Schematic representation of the chemical structures of fatty acids (including palmitic acid, linoleic acid and docosahexaenoic acid), glycerolipids (including diacylglycerol and triacylglycerol), glycerophospholipids (including phosphatidylcholine, lysophosphatidylcholine and lysophosphatidylethanolamine), sterols (including cholesterol and cholesteryl ester) and sphingolipids (including sphingomyelin). Side chains indicated with an R correspond to a fatty acid. Chemical structures are adapted from the LIPID MAPS structure database[1].

Supplementary Data 1. **Detailed results of the PRS-based analyses in phase I.** Results are classified by lipid species (fatty acyls, glyceroplipids, glycerophospholipids, sphingolipids, sterols, lipoproteins and others). For each lipid species, the SNP P-value threshold (P_T; thresh), the P-value (Pval), the variance explained (R^2), and the number of SNPs (nSNPs) are listed; P-values lower than 1.93E-05 (significant after Bonferroni correction) are highlighted in green; R higher than 1% is highlighted in blue.

Fatty acids (FA) - Kettunen et al., 2016 & Shin et al., 2014

LA (Linoleic acid)

thresh	pval	r2	nsnps
0.001	0.138954603	8.70285E-05	1410
0.05	0.082314635	0.000142755	34881
0.1	0.120714396	0.000101439	62719
0.2	0.098572621	0.000122961	111437
0.3	0.238514494	3.73799E-05	154457
0.4	0.237022091	3.78889E-05	192532
0.5	0.269071974	2.8017E-05	226772

DHA (docosahexaenoic acid)

thresh	pval	r2	nsnps
0.001	0.40781981	4.02711E-06	1410
0.05	0.228079616	4.11357E-05	34881
0.1	0.22103042	4.37747E-05	62718
0.2	0.178855331	6.2663E-05	111436
0.3	0.346790944	1.14997E-05	154455
0.4	0.297043581	2.10389E-05	192531
0.5	0.340176617	1.25733E-05	226767

Bis.DB.ratio

thresh	pval	r2	nsnps
0.001	0.01376715	0.000359074	1410
0.05	0.379200579	6.9954E-06	34876
0.1	0.376233767	7.35499E-06	62703
0.2	0.201671398	5.16356E-05	111414
0.3	0.149156935	7.99818E-05	154425
0.4	0.115755721	0.000105847	192495
0.5	0.147869663	8.08388E-05	226729

Bis.FA.ratio

thresh	pval	r2	nsnps
0.001	0.062889046	0.00017794	1407
0.05	0.496942901	4.4585E-09	34798
0.1	0.274219265	2.73417E-05	62572
0.2	0.139368347	8.90735E-05	111170
0.3	0.135451536	9.20289E-05	154087
0.4	0.122055012	0.000103001	192065
0.5	0.146351733	8.40557E-05	226213

DB.in.FA

thresh	pval	r2	nsnps
0.001	0.254491384	2.77309E-05	1411
0.05	0.407478456	3.4825E-06	35002
0.1	0.178249296	5.40544E-05	62933
0.2	0.145361068	7.09712E-05	111837
0.3	0.143781201	7.19073E-05	155070
0.4	0.105959617	9.9071E-05	193298
0.5	0.114860514	9.17168E-05	227676

CH2.in.FA

thresh	pval	r2	nsnps
0.001	0.426354534	2.54735E-06	1411
0.05	0.06939799	0.000161918	35038
0.1	0.042383276	0.000219534	62995
0.2	0.000103937	0.001016224	111967
0.3	0.000160495	0.000956072	155265
0.4	5.28692E-05	0.001110186	193561
0.5	0.000119775	0.000996566	227987

CH2.DB.ratio

thresh	pval	r2	nsnps
0.001	0.166797576	4.91493E-05	1410
0.05	0.299766374	1.44945E-05	34876
0.1	0.129374602	6.70519E-05	62713
0.2	0.024761038	0.000202785	111427
0.3	0.023114915	0.000208863	154449
0.4	0.014750107	0.000249072	192514
0.5	0.020022691	0.000221623	226750

Tot.FA

thresh	pval	r2	nsnps
0.001	0.244045755	3.55959E-05	1410
0.05	0.123383631	9.93296E-05	34877
0.1	0.249695851	3.37816E-05	62710
0.2	0.233363292	3.92229E-05	111418
0.3	0.381108744	6.77885E-06	154435
0.4	0.403242405	4.44331E-06	192496
0.5	0.47712782	2.43656E-07	226735

FALen (fatty acid length)

thresh	pval	r2	nsnps
0.001	0.013625756	0.000361674	1410
0.05	0.046017396	0.000210605	34876
0.1	0.148829607	8.04844E-05	62707
0.2	0.027969114	0.000271104	111412
0.3	0.020078752	0.000312449	154425
0.4	0.016161743	0.000339899	192491
0.5	0.013110299	0.000366615	226728

FAw3

thresh	pval	r2	nsnps
0.001	0.408557591	3.94859E-06	1408
0.05	0.038449087	0.000231018	34866
0.1	0.094559011	0.000127316	62698
0.2	0.054114477	0.000190464	111413
0.3	0.090383022	0.00013225	154428
0.4	0.107477577	0.00011353	192486
0.5	0.158886955	7.36895E-05	226716

FAw6

thresh	pval	r2	nsnps
0.001	0.194552642	5.49175E-05	1409
0.05	0.043818624	0.000215973	34868
0.1	0.073058223	0.000156387	62696
0.2	0.015675727	0.000343019	111386
0.3	0.053806058	0.00019167	154399
0.4	0.048826942	0.000203107	192455
0.5	0.066928359	0.000166371	226690

FAw79S

thresh	pval	r2	nsnps
0.001	0.150422885	7.92536E-05	1410
0.05	0.187337412	5.83502E-05	34877
0.1	0.340406514	1.25286E-05	62712
0.2	0.421264345	2.92209E-06	111419
0.3	0.452332715	1.06211E-06	154437
0.4	0.410503064	3.79013E-06	192500
0.5	0.351276108	1.07974E-05	226738

MUFA

thresh	pval	r2	nsnps
0.001	0.053311152	0.000192341	1410
0.05	0.306776131	1.88424E-05	34882
0.1	0.410135747	3.81366E-06	62720
0.2	0.326426667	1.49479E-05	111441
0.3	0.211370955	4.74817E-05	154463
0.4	0.167772198	6.85149E-05	192531
0.5	0.151944409	7.80945E-05	226771

otPUFA

thresh	pval	r2	nsnps
0.001	0.161648638	7.19998E-05	1410
0.05	0.1346126	9.00904E-05	34875
0.1	0.128854848	9.45426E-05	62710
0.2	0.050379646	0.000198776	111424
0.3	0.089615519	0.000133137	154440
0.4	0.084658785	0.000139408	192509
0.5	0.140000807	8.61342E-05	226742

dihomo-linolenate (20:3n3 or n6)

thresh	pval	r2	nsnps
0.001	0.039970812	0.000416614	868
0.05	0.000187501	0.00171812	20370
0.1	7.55E-06	0.00254175	36492
0.2	4.12E-07	0.003297056	64866
0.3	1.64E-06	0.002938062	89586
0.4	5.32E-06	0.002632261	111586
0.5	2.68E-06	0.002809512	131214

docosapentaenoate (n3 DPA; 22:5n3)

thresh	pval	r2	nsnps
0.001	0.098888463	0.000224946	868
0.05	0.003832942	0.000963853	20370
0.1	0.000261309	0.001630729	36492
0.2	0.00024536	0.001646629	64867
0.3	0.000294251	0.001600779	89562
0.4	0.002421787	0.001075908	111586
0.5	0.002956229	0.001027112	131212

eicosapentaenoate (EPA; 20:5n3)

thresh	pval	r2	nsnps
0.001	0.260078568	5.61E-05	868
0.05	1.46E-05	0.002367868	20371
0.1	1.55E-06	0.002949213	36493
0.2	1.47E-07	0.003562566	64867
0.3	2.19E-09	0.004664429	89564
0.4	2.79E-08	0.003996741	111587
0.5	1.10E-07	0.003638742	131216

linoleate (18:2n6)

thresh	pval	r2	nsnps
0.001	0.188546854	0.000106384	868
0.05	0.030410578	0.000479205	20370
0.1	0.002454182	0.001078498	36492
0.2	0.004875955	0.00091049	64867
0.3	0.00656724	0.000838446	89561
0.4	0.009627793	0.000746776	111584
0.5	0.00891362	0.000765157	131215

linolenate [alpha or gamma; (18:3n3 or 6)]

thresh	pval	r2	nsnps
0.001	0.152204433	0.000143732	868
0.05	0.002102169	0.001115946	20370
0.1	7.82E-05	0.00194593	36492
0.2	0.001020455	0.001295477	64867
0.3	0.000366626	0.001552761	89562
0.4	0.00069594	0.001391308	111586
0.5	0.00118242	0.001258723	131212

Docosapentaenoic acid (n6-DPA)

thresh	pval	r2	nsnps
0.001	0.059328585	0.000956303	869
0.05	0.000681189	0.004021769	20371
0.1	0.008898413	0.002204262	36493
0.2	0.006561804	0.002414515	64864
0.3	0.009770529	0.002140118	89584
0.4	0.033205651	0.001323118	111583
0.5	0.054201771	0.001012196	131210

Stearamide

thresh	pval	r2	nsnps
0.001	0.009641205	0.002210859	868
0.05	0.081535498	0.000786075	20371
0.1	0.004449593	0.002762123	36492
0.2	0.002554033	0.003164254	64866
0.3	0.000832139	0.003988489	89561

Isovalerate

thresh	pval	r2	nsnps
0.001	0.246226128	7.00E-05	869
0.05	0.182534879	0.000121801	20369
0.1	0.422086677	5.74E-06	36493
0.2	0.34180136	2.47E-05	64868
0.3	0.442504578	3.11E-06	89559

Butyrylcarnitine

thresh	pval	r2	nsnps
0.001	0.138562358	0.000160708	868
0.05	0.034001788	0.000453097	20370
0.1	0.0552626	0.000346476	36492
0.2	0.061856359	0.000322397	64868
0.3	0.096475635	0.000230609	89565

15-methylpalmitate

thresh	pval	r2	nsnps
0.001	0.407820931	7.83E-06	868
0.05	0.122011576	0.000195487	20371
0.1	0.059611541	0.000349622	36493
0.2	0.091347523	0.000255738	64867
0.3	0.127306277	0.000186934	89563

0.4	0.004369369	0.002775227	111585
0.5	0.002585694	0.003155281	131211

2-hydroxyglutarate

thresh	pval	r2	nsnps
0.001	0.317013577	3.82E-05	869
0.05	0.029519052	0.000600663	20364
0.1	0.012713604	0.000841614	36484
0.2	0.019129623	0.000723518	64852
0.3	0.057268904	0.000419748	89548
0.4	0.022667041	0.000675121	111560
0.5	0.01309687	0.000832961	131186

octadecanedioate

thresh	pval	r2	nsnps
0.001	0.037673999	0.000459826	869
0.05	0.336786824	2.58E-05	20371
0.1	0.248107423	6.73E-05	36493
0.2	0.327513084	2.90E-05	64863
0.3	0.306803378	3.71E-05	89590
0.4	0.224180365	8.36E-05	111594
0.5	0.254426656	6.35E-05	131212

2-hydroxystearate

thresh	pval	r2	nsnps
0.001	0.469763927	7.87E-07	868
0.05	0.005432947	0.000886431	20370
0.1	0.000944711	0.00131888	36492
0.2	0.000122112	0.001837816	64867
0.3	0.001318509	0.001235464	89589
0.4	0.002719704	0.001055797	111589
0.5	0.004231949	0.000947274	131217

caproate (6:0)

thresh	pval	r2	nsnps
0.001	0.4525867	1.93E-06	868
0.05	0.395415273	9.55E-06	20371
0.1	0.179413935	0.000114348	36493
0.2	0.133554263	0.000167246	64868
0.3	0.260452062	5.60E-05	89588
0.4	0.435491177	3.58E-06	111587
0.5	0.420922361	5.41E-06	131214

pelargonate (9:0)

thresh	pval	r2	nsnps
0.001	0.499884266	1.14E-11	868
0.05	0.353729384	1.91E-05	20370
0.1	0.463219219	1.16E-06	36492
0.2	0.184572627	0.000109638	64866
0.3	0.138104688	0.000161168	89563
0.4	0.134873535	0.00016557	111585
0.5	0.12247785	0.000183758	131211

adrenate (22:4n6)

thresh	pval	r2	nsnps
0.001	0.054827251	0.000349079	868
0.05	0.372717479	1.44E-05	20371
0.1	0.178684396	0.000115562	36493
0.2	0.092984765	0.000238616	64868
0.3	0.241887585	6.69E-05	89587
0.4	0.293721088	4.02E-05	111587
0.5	0.277341298	4.76E-05	131214

0.4	0.46927751	8.82E-07	111587
0.5	0.439424692	3.45E-06	131205

3-carboxy-4-methyl-5-propyl-2-furanpropanoate (CMPF)

thresh	pval	r2	nsnps
0.001	0.05608581	0.000342649	868
0.05	0.311072896	3.30E-05	20370
0.1	0.351521142	1.97E-05	36492
0.2	0.287405051	4.27E-05	64868
0.3	0.308008932	3.42E-05	89563
0.4	0.287399532	4.27E-05	111584
0.5	0.329251588	2.65E-05	131214

tetradecanedioate

thresh	pval	r2	nsnps
0.001	0.388604498	1.42E-05	870
0.05	0.259481054	7.39E-05	20362
0.1	0.227144532	9.95E-05	36496
0.2	0.101651183	0.000287486	64872
0.3	0.098924968	0.000294534	89564
0.4	0.09674912	0.000300324	111573
0.5	0.050976086	0.000475058	131207

valerate

thresh	pval	r2	nsnps
0.001	0.128133439	0.000321829	868
0.05	0.207971752	0.000165258	20367
0.1	0.390590733	1.93E-05	36486
0.2	0.347773926	3.82E-05	64856
0.3	0.317691688	5.62E-05	89542
0.4	0.324607418	5.17E-05	111567
0.5	0.314892292	5.80E-05	131191

caprylate (8:0)

thresh	pval	r2	nsnps
0.001	0.417677485	5.87E-06	868
0.05	0.304808944	3.54E-05	20371
0.1	0.227133309	7.61E-05	36493
0.2	0.081283169	0.000265116	64868
0.3	0.086778975	0.000251762	89564
0.4	0.105768841	0.000212197	111587
0.5	0.097441308	0.000228433	131214

undecanoate (11:0)

thresh	pval	r2	nsnps
0.001	0.072955154	0.000298571	868
0.05	0.214305392	8.85E-05	20368
0.1	0.42386826	5.21E-06	36489
0.2	0.162268166	0.000137058	64861
0.3	0.049372909	0.000384855	89578
0.4	0.030582546	0.000494936	111578
0.5	0.027695335	0.000518194	131204

arachidonate (20:4n6)

thresh	pval	r2	nsnps
0.001	0.144262266	0.000152899	868
0.05	0.0011593	0.001258674	20370
0.1	0.000415292	0.00151529	36492
0.2	4.96E-06	0.002647021	64867
0.3	4.51E-06	0.002671848	89585
0.4	3.07E-05	0.002177366	111584
0.5	5.83E-05	0.002013271	131212

0.4	0.129453832	0.000173422	111587
0.5	0.240998591	6.73E-05	131213

dodecanedioate

thresh	pval	r2	nsnps
0.001	0.480021261	4.14E-07	870
0.05	0.418740296	6.94E-06	20362
0.1	0.325451988	3.38E-05	36476
0.2	0.415264863	7.55E-06	64871
0.3	0.478006598	5.02E-07	89585
0.4	0.423089577	6.20E-06	111600
0.5	0.475966794	5.99E-07	131220

n-Butyl Oleate

thresh	pval	r2	nsnps
0.001	0.053336165	0.000602747	870
0.05	0.000379051	0.002623633	20362
0.1	0.003094494	0.001734523	36486
0.2	0.000737195	0.002339245	64854
0.3	0.000828124	0.002289742	89540
0.4	0.000467485	0.002533757	111560
0.5	0.000387241	0.002614462	131185

10-undecenoate (11:1n1)

thresh	pval	r2	nsnps
0.001	0.081083863	0.000265512	868
0.05	0.011415979	0.000703917	20370
0.1	0.004700652	0.000916263	36492
0.2	0.001492781	0.001197471	64867
0.3	0.000607897	0.001421412	89564
0.4	0.000587938	0.001429783	111588
0.5	0.000455794	0.001493725	131215

heptanoate (7:0)

thresh	pval	r2	nsnps
0.001	0.31565768	3.13E-05	868
0.05	0.314308205	3.18E-05	20370
0.1	0.445700846	2.53E-06	36492
0.2	0.191718031	0.000103279	64867
0.3	0.134342548	0.000166328	89587
0.4	0.181702968	0.00011231	111586
0.5	0.152886879	0.000142593	131213

10-heptadecenoate (17:1n7)

thresh	pval	r2	nsnps
0.001	0.139459089	0.000159513	868
0.05	0.058705505	0.00033353	20370
0.1	0.013151413	0.000671431	36492
0.2	0.031043046	0.000473526	64867
0.3	0.027379005	0.000501907	89559
0.4	0.044712252	0.000392453	111583
0.5	0.040355675	0.000415007	131211

dihomo-linoleate (20:2n6)

thresh	pval	r2	nsnps
0.001	0.036055682	0.000439764	868
0.05	0.063155702	0.000317815	20370
0.1	0.019665498	0.000577354	36492
0.2	0.041420829	0.000409035	64867
0.3	0.064884084	0.000312106	89562
0.4	0.129939734	0.000172622	111586
0.5	0.140043484	0.000158653	131213

0.4	0.143286621	0.000163578	111588
0.5	0.118997345	0.000200561	131216

hexadecanedioate

thresh	pval	r2	nsnps
0.001	0.318776121	3.44E-05	869
0.05	0.399887172	9.98E-06	20366
0.1	0.415993802	6.98E-06	36499
0.2	0.350665789	2.28E-05	64885
0.3	0.260671543	6.38E-05	89626
0.4	0.227233755	8.68E-05	111632
0.5	0.235303351	8.07E-05	131266

2-hydroxypalmitate

thresh	pval	r2	nsnps
0.001	0.155603855	0.000139558	868
0.05	0.001647462	0.001174664	20370
0.1	5.53E-05	0.002032257	36492
0.2	3.89E-05	0.002122308	64867
0.3	0.000197605	0.001707129	89563
0.4	0.000119953	0.001834118	111584
0.5	0.000172805	0.001741196	131211

5-dodecenoate (12:1n7)

thresh	pval	r2	nsnps
0.001	0.159300835	0.000135839	869
0.05	0.028568198	0.000494089	20370
0.1	0.011362653	0.000708487	36490
0.2	0.024406955	0.000530007	64868
0.3	0.01707333	0.00061267	89565
0.4	0.05362138	0.000354265	111587
0.5	0.029952365	0.00048336	131213

laurate (12:0)

thresh	pval	r2	nsnps
0.001	0.154710945	0.00014063	868
0.05	0.017442243	0.0006057	20371
0.1	0.008189147	0.000784038	36492
0.2	0.015918514	0.000626982	64866
0.3	0.019042343	0.000585349	89562
0.4	0.036186904	0.000439373	111585
0.5	0.027081677	0.000504591	131213

10-nonadecenoate (19:1n9)

thresh	pval	r2	nsnps
0.001	0.039423282	0.000420404	868
0.05	0.005634432	0.000874011	20371
0.1	0.002670138	0.001056004	36493
0.2	0.01126101	0.000708394	64868
0.3	0.010454226	0.000726	89563
0.4	0.018894408	0.000587234	111586
0.5	0.016300311	0.00062154	131214

eicosenoate (20:1n9 or 11)

thresh	pval	r2	nsnps
0.001	0.160312471	0.000134158	868
0.05	0.138831222	0.000160283	20370
0.1	0.036092459	0.000439597	36492
0.2	0.075141831	0.000281431	64867
0.3	0.045460967	0.000388659	89562
0.4	0.071617781	0.000291422	111586
0.5	0.094351529	0.000234972	131212

margarate (17:0)

thresh	pval	r2	nsnps
0.001	0.132435534	0.00016914	868
0.05	0.000229543	0.00166889	20370
0.1	3.47E-05	0.00215148	36492
0.2	0.001074958	0.001280568	64867
0.3	0.000361475	0.00155401	89562
0.4	0.00075272	0.001369581	111586
0.5	0.000423618	0.001513996	131212

myristate (14:0)

thresh	pval	r2	nsnps
0.001	0.240062505	6.77E-05	868
0.05	0.015041246	0.00063883	20370
0.1	0.003942296	0.000958451	36492
0.2	0.01344277	0.000665107	64867
0.3	0.012875392	0.000675224	89562
0.4	0.016017911	0.000624171	111586
0.5	0.013369976	0.00066638	131212

myristoleate (14:1n5)

thresh	pval	r2	nsnps
0.001	0.064302825	0.000313922	868
0.05	0.018196932	0.000595139	20370
0.1	0.001052638	0.001284749	36492
0.2	0.00105128	0.001285071	64867
0.3	0.000228092	0.001669136	89564
0.4	0.00055757	0.001443676	111587
0.5	0.000400465	0.00152692	131213

nonadecanoate (19:0)

thresh	pval	r2	nsnps
0.001	0.450337514	2.12E-06	868
0.05	0.02775065	0.000499802	20370
0.1	0.025120863	0.000522477	36490
0.2	0.066509664	0.000307643	64864
0.3	0.095656154	0.000232759	89558
0.4	0.12576511	0.000179232	111582
0.5	0.132636652	0.000169178	131210

oleate (18:1n9)

thresh	pval	r2	nsnps
0.001	0.091553068	0.00024198	868
0.05	0.121687576	0.000185827	20369
0.1	0.060459559	0.000328415	36491
0.2	0.205175339	9.26E-05	64866
0.3	0.141531752	0.000157344	89560
0.4	0.214709662	8.53E-05	111582
0.5	0.221069374	8.07E-05	131210

palmitate (16:0)

thresh	pval	r2	nsnps
0.001	0.276087006	4.81E-05	868
0.05	0.076016879	0.000279032	20371
0.1	0.0471841	0.000380536	36493
0.2	0.138582926	0.000160615	64868
0.3	0.126181635	0.000178191	89563
0.4	0.185781782	0.000108594	111586
0.5	0.162165718	0.000132118	131214

palmitoleate (16:1n7)

thresh	pval	r2	nsnps
0.001	0.117705145	0.00019213	868
0.05	0.195275224	0.000100613	20370
0.1	0.083812537	0.000259835	36492
0.2	0.09301454	0.000238649	64867
0.3	0.071928935	0.000291509	89559
0.4	0.096635825	0.000230968	111583
0.5	0.114982428	0.000196661	131211

pentadecanoate (15:0)

thresh	pval	r2	nsnps
0.001	0.368989997	1.58E-05	868
0.05	0.011628815	0.000728767	20367
0.1	0.001313173	0.001280564	36497
0.2	0.007655114	0.000832219	64871
0.3	0.012221446	0.000716567	89566
0.4	0.01322866	0.000697177	111592
0.5	0.00891723	0.000794295	131220

stearate (18:0)

thresh	pval	r2	nsnps
0.001	0.335879536	2.44E-05	868
0.05	0.016809292	0.000613544	20371
0.1	0.016026374	0.000624641	36493
0.2	0.048408189	0.000374807	64868
0.3	0.059465486	0.000330515	89562
0.4	0.138412204	0.000160778	111584
0.5	0.107342876	0.000209297	131212

stearidonate (18:4n3)

thresh	pval	r2	nsnps
0.001	0.110235927	0.000204794	868
0.05	8.56E-05	0.001924812	20370
0.1	1.12E-06	0.003047176	36492
0.2	2.78E-06	0.002811074	64865
0.3	1.34E-07	0.003603613	89563
0.4	6.73E-07	0.003181429	111585
0.5	1.52E-06	0.002968563	131212

5,8-tetradecadienoate

thresh	pval	r2	nsnps
0.001	0.162032227	0.000132589	868
0.05	0.000990658	0.00130359	20368
0.1	6.24E-05	0.002004894	36490
0.2	0.000336688	0.00157516	64865
0.3	0.000608888	0.00142565	89561
0.4	0.002791991	0.001046613	111584
0.5	0.00091232	0.001324195	131209

12-hydroxyeicosatetraenoate (12-HETE)

thresh	pval	r2	nsnps
0.001	0.051173206	0.000978821	868
0.05	0.206724368	0.000245424	20368
0.1	0.299997298	0.000100914	36491
0.2	0.270725195	0.000136819	64870
0.3	0.142264515	0.000420216	89590
0.4	0.290632831	0.000111624	111592
0.5	0.311436643	8.87E-05	131216

Acylcarnitines (AC) - Draisma et al., 2015 & Shin et al., 2014

AC 0

thresh	pval	r2	nsnps
0.001	0.033316504	0.000449762	939
0.05	0.000174021	0.001709201	22496
0.1	0.001053355	0.001263464	40453
0.2	0.001843982	0.001126858	72151
0.3	0.000248284	0.001620578	100010
0.4	0.000241491	0.001627485	124958
0.5	0.00063878	0.001386397	147254

AC 2:0

thresh	pval	r2	nsnps
0.001	0.219359293	8.01829E-05	939
0.05	0.002683403	0.001036036	22495
0.1	8.35409E-05	0.001892993	40459
0.2	6.72965E-06	0.002530128	72209
0.3	2.32174E-05	0.002215832	100024
0.4	2.45817E-05	0.002201385	125002
0.5	9.99919E-05	0.001847881	147262

AC 3:0

thresh	pval	r2	nsnps
0.001	0.001802628	0.005321214	819
0.05	0.00285219	0.004800402	18895
0.1	0.010788263	0.003319173	33882
0.2	0.068519409	0.001391285	60105
0.3	0.05982337	0.001523988	83131
0.4	0.027788283	0.002305086	103642
0.5	0.014591878	0.002990947	121933

AC 3:0 OH

thresh	pval	r2	nsnps
0.001	0.342646887	0.000103347	802
0.05	0.07538762	0.001298332	18810
0.1	0.024173718	0.002450026	33773
0.2	0.044134107	0.001826949	60006
0.3	0.014141373	0.003022954	82923
0.4	0.007171533	0.003765968	103399
0.5	0.005226441	0.00411739	121739

AC 5:1 DC

thresh	pval	r2	nsnps
0.001	0.237089141	0.000239776	837
0.05	0.493446673	1.2634E-07	19354
0.1	0.331029568	8.94304E-05	34808
0.2	0.272764325	0.000171047	61838
0.3	0.210631966	0.000302757	85614
0.4	0.329902266	9.0708E-05	106872
0.5	0.402653989	2.84425E-05	125735

AC 7:0 DC

thresh	pval	r2	nsnps
0.001	0.073327078	0.000365809	933
0.05	0.000884228	0.001696297	22349
0.1	0.000199371	0.002175138	40154
0.2	6.41983E-05	0.002543682	71704
0.3	0.000171901	0.002223172	99352
0.4	4.043E-05	0.002694882	124180
0.5	4.50925E-05	0.002659156	146327

AC 8:0

thresh	pval	r2	nsnps
0.001	0.470254028	2.03874E-06	826
0.05	0.087337644	0.000674134	19284
0.1	0.046951858	0.001026613	34617
0.2	0.004387727	0.002510739	61609
0.3	0.000786382	0.00365048	85258
0.4	0.00035082	0.004195018	106394
0.5	0.000633385	0.003795973	125250

AC 8:1

thresh	pval	r2	nsnps
0.001	0.102665366	0.000214479	938
0.05	0.000890518	0.00130465	22495
0.1	0.000141663	0.001760622	40457
0.2	1.13522E-05	0.002397205	72189
0.3	0.000167476	0.001718777	100035
0.4	0.000211512	0.001660515	125027
0.5	0.00024111	0.001627878	147292

AC 9:0

thresh	pval	r2	nsnps
0.001	0.323965538	4.57066E-05	897
0.05	0.026212387	0.000824334	21243
0.1	0.028644801	0.000791918	38165
0.2	0.021537897	0.000896666	67966
0.3	0.005722273	0.001400804	94074
0.4	0.010682363	0.001160394	117505
0.5	0.020865593	0.000908415	138345

AC 10:0

thresh	pval	r2	nsnps
0.001	0.476199845	1.30381E-06	841
0.05	0.089386797	0.000661248	19280
0.1	0.062527731	0.000860528	34630
0.2	0.0099656	0.00198044	61625
0.3	0.002236762	0.002952405	85261
0.4	0.000778514	0.003655898	106375
0.5	0.00103158	0.003467256	125216

AC 10:1

thresh	pval	r2	nsnps
0.001	0.017897393	0.000765451	940
0.05	0.051634497	0.000461113	22347
0.1	0.007478353	0.001028413	40165
0.2	0.005571415	0.001118817	71711
0.3	0.005623013	0.001115975	99336
0.4	0.002800294	0.001332651	124147
0.5	0.004383754	0.00119296	146279

AC 10:2

thresh	pval	r2	nsnps
0.001	0.382973107	1.53923E-05	938
0.05	0.158186091	0.000174361	22343
0.1	0.474421945	7.15014E-07	40171
0.2	0.277344824	6.0617E-05	71661
0.3	0.463847295	1.4304E-06	99271
0.4	0.400640185	1.10036E-05	124090
0.5	0.42365704	6.43982E-06	146234

AC 12:1

thresh	pval	r2	nsnps
0.001	0.225956115	7.56659E-05	940
0.05	0.305909956	3.44382E-05	22494
0.1	0.22791112	7.43665E-05	40453
0.2	0.246260661	6.29846E-05	72199
0.3	0.331051295	2.55385E-05	100028
0.4	0.217346563	8.16029E-05	124980
0.5	0.245605102	6.33671E-05	147267

AC 14:1

thresh	pval	r2	nsnps
0.001	0.083813716	0.00025462	937
0.05	0.007813964	0.000781327	22483
0.1	0.023965261	0.000523124	40455
0.2	0.009279492	0.000741012	72184
0.3	0.001005959	0.001274916	100020
0.4	0.001303804	0.001211451	125005
0.5	0.000767039	0.001341513	147295

AC 14:1 OH

thresh	pval	r2	nsnps
0.001	0.011596792	0.002820097	819
0.05	0.34493891	8.72387E-05	18924
0.1	0.125903698	0.000719329	33867
0.2	0.084985106	0.001031361	60115
0.3	0.141117726	0.000633385	83126
0.4	0.11513235	0.000788306	103595
0.5	0.100442034	0.000896001	121840

AC 14:2

thresh	pval	r2	nsnps
0.001	0.007645919	0.001021283	933
0.05	3.03739E-06	0.003548404	22348
0.1	5.22911E-10	0.006447342	40178
0.2	1.7651E-13	0.009144756	71669
0.3	7.70674E-14	0.009424936	99306
0.4	1.60549E-15	0.010734494	124131
0.5	2.19894E-15	0.010628057	146256

AC 18:1

thresh	pval	r2	nsnps
0.001	0.252109337	6.75191E-05	939
0.05	0.455448369	1.89574E-06	22436
0.1	0.136229401	0.000182291	40355
0.2	0.034751902	0.000498619	72012
0.3	0.032294087	0.000516891	99786
0.4	0.024340645	0.000588127	124694
0.5	0.029210667	0.000542033	146946

AC 18:2

thresh	pval	r2	nsnps
0.001	0.055540833	0.00033944	939
0.05	0.06351261	0.00031133	22484
0.1	0.02975524	0.000474743	40444
0.2	0.010433519	0.000713554	72168
0.3	0.006545513	0.000822979	100004
0.4	0.006905683	0.000810329	124991
0.5	0.015646692	0.000619866	147267

3-dehydrocarnitine

thresh	pval	r2	nsnps
0.001	0.330756936	2.60E-05	868
0.05	0.055401083	0.000345375	20370
0.1	0.067499522	0.000303453	36492
0.2	0.058661453	0.000333147	64866
0.3	0.018150491	0.000595246	89560
0.4	0.014491161	0.000647618	111585
0.5	0.019060598	0.000583936	131211

AC 6:0

thresh	pval	r2	nsnps
0.001	0.434032153	3.76E-06	868
0.05	0.17326868	0.000120714	20369
0.1	0.147158584	0.000149821	36491
0.2	0.16880371	0.000125264	64864
0.3	0.02800675	0.000497377	89582
0.4	0.017742607	0.000602226	111582
0.5	0.030086073	0.000481169	131210

AC 12:0

thresh	pval	r2	nsnps
0.001	0.102985027	0.000324725	869
0.05	0.385814891	1.71E-05	20368
0.1	0.329600173	3.95E-05	36491
0.2	0.444137066	4.01E-06	64878
0.3	0.325456698	4.16E-05	89604
0.4	0.197918621	0.000146372	111607
0.5	0.272943421	7.41E-05	131231

AC 16:0

thresh	pval	r2	nsnps
0.001	0.308178957	3.46E-05	868
0.05	0.455016566	1.76E-06	20368
0.1	0.31212147	3.31E-05	36491
0.2	0.326727262	2.78E-05	64868
0.3	0.481703424	2.90E-07	89558
0.4	0.270524086	5.15E-05	111587
0.5	0.322514328	2.92E-05	131213

AC 18:0

thresh	pval	r2	nsnps
0.001	0.357702968	1.96E-05	869
0.05	0.005492914	0.000955159	20368
0.1	0.000775572	0.001478937	36489
0.2	3.92E-05	0.002301694	64864
0.3	5.89E-05	0.002188491	89555
0.4	1.11E-06	0.003304413	111588
0.5	2.53E-06	0.003071113	131212

Glycerophospholipids metabolism - Shin et al., 2014

Glycerol 3-phosphate (G3P)

thresh	pval	r2	nsnps
0.001	0.324870564	2.81E-05	869
0.05	0.077804675	0.000274786	20369
0.1	0.002044389	0.001122984	36491
0.2	0.001371654	0.00122193	64865
0.3	0.005389355	0.000885725	89584
0.4	0.006734961	0.000831918	111584
0.5	0.007793852	0.000796847	131211

Glycerophosphorylcholine (GPC)

thresh	pval	r2	nsnps
0.001	0.018015092	0.000647929	868
0.05	0.30859204	3.68E-05	20370
0.1	0.306744404	3.76E-05	36491
0.2	0.181116999	0.000122395	64868
0.3	0.227972311	8.20E-05	89567
0.4	0.332453697	2.77E-05	111590
0.5	0.496358544	1.23E-08	131220

Monoacylglycerol (MAG) - Shin et al., 2014

1-linoleoylglycerol

thresh	pval	r2	nsnps
0.001	0.073103925	0.000764976	868
0.05	0.438578137	8.66E-06	20369
0.1	0.422528045	1.38E-05	36490
0.2	0.450349418	5.64E-06	64864
0.3	0.338177805	6.32E-05	89558
0.4	0.263770481	0.000144653	111580
0.5	0.256513711	0.000155076	131208

1-oleoylglycerol (MAG 18:1)

thresh	pval	r2	nsnps
0.001	0.40315649	1.11E-05	869
0.05	3.22E-08	0.005362258	20371
0.1	2.42E-09	0.006281556	36495
0.2	4.70E-09	0.006045121	64876
0.3	5.04E-09	0.006020721	89589
0.4	1.08E-09	0.006569361	111589
0.5	1.35E-08	0.005670375	131216

1-palmitoylglycerol

thresh	pval	r2	nsnps
0.001	0.008669214	0.000808615	867
0.05	0.070039054	0.00031101	20363
0.1	0.214714205	8.92E-05	36487
0.2	0.29811268	4.01E-05	64869
0.3	0.301656034	3.86E-05	89566
0.4	0.308270138	3.58E-05	111588
0.5	0.294261263	4.18E-05	131212

1-stearoylglycerol

thresh	pval	r2	nsnps
0.001	0.319882093	3.34E-05	869
0.05	0.154894862	0.000157335	20372
0.1	0.041866041	0.000456109	36496
0.2	0.005935524	0.000965066	64873
0.3	0.001151744	0.001416091	89595
0.4	0.002401032	0.001212278	111598
0.5	0.002247686	0.001230485	131221

Diacylglycerol (DAG) - Rhee et al., 2013

DAG 34:1

thresh	pval	r2	nsnps
0.001	0.03466996	0.001587648	962
0.05	0.211885045	0.000308159	23182
0.1	0.490371671	2.80633E-07	41762
0.2	0.352511407	6.90243E-05	74775
0.3	0.387575276	3.93021E-05	103797
0.4	0.206551333	0.000322613	129896
0.5	0.218473051	0.000291041	153317

DAG 34:2

thresh	pval	r2	nsnps
0.001	0.011659869	0.002475029	962
0.05	0.094936535	0.000827501	23182
0.1	0.25003264	0.00021905	41762
0.2	0.346480063	7.50933E-05	74775
0.3	0.353316635	6.82361E-05	103797
0.4	0.497803135	1.46071E-08	129896
0.5	0.493380323	1.32637E-07	153317

DAG 36:1

thresh	pval	r2	nsnps
0.001	0.243505182	0.000232692	962
0.05	0.294793295	0.000140159	23182
0.1	0.329014861	9.43721E-05	41762
0.2	0.148839556	0.000522293	74775
0.3	0.104137999	0.000762411	103797
0.4	0.033762493	0.001608619	129896
0.5	0.040989655	0.001456173	153317

DAG 36:2

thresh	pval	r2	nsnps
0.001	0.00182665	0.00406137	962
0.05	0.068250779	0.00106733	23182
0.1	0.164043276	0.000460605	41762
0.2	0.142528618	0.00055034	74775
0.3	0.176331216	0.000416028	103797
0.4	0.304163471	0.000126494	129896
0.5	0.332571724	9.02347E-05	153317

Triacylglycerol (TAG) - Rhee et al., 2013

TAG 44:1

thresh	pval	r2	nsnps
0.001	0.398221875	3.20512E-05	962
0.05	0.044601712	0.001390502	23182
0.1	0.000246738	0.005830795	41762
0.2	0.000155401	0.006244463	74775
0.3	2.49247E-05	0.007894412	103797
0.4	2.01276E-06	0.01018509	129896
0.5	9.18986E-06	0.008800272	153317

TAG 46:0

thresh	pval	r2	nsnps
0.001	0.140644717	0.00055902	962
0.05	0.018783833	0.00208092	23182
0.1	0.000345301	0.005531069	41762
0.2	0.000200776	0.006015053	74775
0.3	0.000377513	0.005451683	103797
0.4	0.000214616	0.005955435	129896
0.5	0.000898959	0.004683218	153317

TAG 46:1

thresh	pval	r2	nsnps
0.001	0.234401102	0.000252761	962
0.05	0.039913268	0.001476957	23182
0.1	0.000555939	0.005107973	41762
0.2	0.000583685	0.005064823	74775
0.3	0.000125481	0.006436302	103797
0.4	1.37374E-05	0.008434839	129896
0.5	5.35224E-05	0.007203316	153317

TAG 46:2

thresh	pval	r2	nsnps
0.001	0.193372044	0.000360799	962
0.05	0.045025419	0.001383178	23171
0.1	0.000202782	0.006006158	41739
0.2	0.000133232	0.006382506	74736
0.3	2.49241E-05	0.007894431	103744
0.4	1.62276E-06	0.010381976	129829
0.5	6.15463E-06	0.009165212	153243

TAG 48:0

thresh	pval	r2	nsnps
0.001	0.460037339	4.84972E-06	962
0.05	0.017273698	0.002149616	23182
0.1	0.001035165	0.004558995	41762
0.2	0.000283447	0.005706995	74775
0.3	7.80756E-05	0.006862924	103797
0.4	1.91421E-05	0.008133702	129896
0.5	4.80431E-05	0.007300815	153317

TAG 48:1

thresh	pval	r2	nsnps
0.001	0.223393952	0.000278761	962
0.05	0.036401029	0.001549225	23182
0.1	0.001434473	0.004272635	41762
0.2	0.000845186	0.0047376	74775
0.3	0.000402841	0.005393926	103797
0.4	5.73818E-05	0.007140491	129896
0.5	0.000185473	0.006086002	153317

TAG 48:2

thresh	pval	r2	nsnps
0.001	0.074319884	0.001004387	962
0.05	0.074072693	0.001006836	23182
0.1	0.0023102	0.003856866	41762
0.2	0.002045313	0.003962812	74775
0.3	0.001075728	0.00452519	103797
0.4	0.000137483	0.006354319	129896
0.5	0.000433306	0.005329125	153317

TAG 48:3

thresh	pval	r2	nsnps
0.001	0.034770334	0.001585365	962
0.05	0.121612907	0.000655758	23182
0.1	0.002755319	0.003703983	41762
0.2	0.004668042	0.003249707	74775
0.3	0.001907411	0.004023637	103797
0.4	0.000315067	0.005612698	129896
0.5	0.00074866	0.004844638	153317

TAG 48:4

thresh	pval	r2	nsnps
0.001	0.042565115	0.00142679	962
0.05	0.46842148	3.02443E-06	23182
0.1	0.050005778	0.001302311	41762
0.2	0.031602436	0.001661059	74775
0.3	0.04338706	0.001411923	103797
0.4	0.021225496	0.001981239	129896
0.5	0.049402228	0.001311632	153317

TAG 50:1

thresh	pval	r2	nsnps
0.001	0.194328593	0.000357903	962
0.05	0.31748978	0.000108553	23182
0.1	0.095787251	0.000821179	41762
0.2	0.075250475	0.000995249	74775
0.3	0.088297842	0.00087921	103797
0.4	0.062819913	0.001129211	129896
0.5	0.11315019	0.000704937	153317

TAG 50:2

thresh	pval	r2	nsnps
0.001	0.106894378	0.000744223	962
0.05	0.027805553	0.001763216	23182
0.1	0.001327211	0.004340734	41762
0.2	0.001148251	0.004467849	74775
0.3	0.000338109	0.005549813	103797
0.4	4.21575E-05	0.00741887	129896
0.5	0.000138918	0.006345007	153317

TAG 50:3

thresh	pval	r2	nsnps
0.001	0.006523313	0.00296418	962
0.05	0.202352092	0.000334387	23182
0.1	0.012680301	0.002405144	41762
0.2	0.020404273	0.002013359	74775
0.3	0.013402071	0.002359151	103797
0.4	0.002841747	0.003677235	129896
0.5	0.005951753	0.003042177	153317

TAG 50:4

thresh	pval	r2	nsnps
0.001	0.006266615	0.002998305	962
0.05	0.482887992	8.8679E-07	23182
0.1	0.069899433	0.001049628	41762
0.2	0.123554811	0.000645057	74775
0.3	0.112435345	0.000709293	103797
0.4	0.047624096	0.001339833	129896
0.5	0.08605345	0.000897716	153317

TAG 50:5

thresh	pval	r2	nsnps
0.001	0.036143836	0.001554809	962
0.05	0.45987557	4.8892E-06	23182
0.1	0.057837104	0.001191455	41762
0.2	0.056650089	0.001207158	74775
0.3	0.074156619	0.001006003	103797
0.4	0.045443958	0.001376016	129896
0.5	0.117584296	0.000678635	153317

TAG 52:1

thresh	pval	r2	nsnps
0.001	0.096687888	0.000814557	962
0.05	0.31538083	0.00011128	23182
0.1	0.131735407	0.000602144	41762
0.2	0.164093445	0.000460414	74775
0.3	0.145657003	0.000536243	103797
0.4	0.070053319	0.001047999	129896
0.5	0.10825562	0.000735445	153317

TAG 52:2

thresh	pval	r2	nsnps
0.001	0.035547758	0.001567914	962
0.05	0.470869251	2.57293E-06	23182
0.1	0.416943866	2.11855E-05	41762
0.2	0.495228221	6.89182E-08	74775
0.3	0.45960884	4.95464E-06	103797
0.4	0.348757956	7.27666E-05	129896
0.5	0.414552402	2.24425E-05	153317

TAG 52:3

thresh	pval	r2	nsnps
0.001	0.002730702	0.003711758	962
0.05	0.024376921	0.001869065	23182
0.1	0.100438885	0.000787734	41762
0.2	0.044718165	0.001388481	74775
0.3	0.06339873	0.001122338	103797
0.4	0.108711711	0.000732533	129896
0.5	0.074276232	0.001004819	153317

TAG 52:4

thresh	pval	r2	nsnps
0.001	0.012036385	0.00244853	962
0.05	0.122853301	0.000648899	23182
0.1	0.297430534	0.000136222	41762
0.2	0.23384663	0.000254024	74775
0.3	0.166422372	0.000451631	103797
0.4	0.242525718	0.000234792	129896
0.5	0.143023154	0.000548085	153317

TAG 52:5

thresh	pval	r2	nsnps
0.001	0.00494921	0.003199633	962
0.05	0.031410987	0.00166589	23182
0.1	0.154279132	0.000499315	41762
0.2	0.115962896	0.000688112	74775
0.3	0.067887605	0.001071294	103797
0.4	0.101542796	0.000780064	129896
0.5	0.055772399	0.001219005	153317

TAG 52:6

thresh	pval	r2	nsnps
0.001	0.013739856	0.002338502	962
0.05	0.009296757	0.002664722	23182
0.1	0.082626116	0.000927072	41762
0.2	0.116487132	0.00068503	74775
0.3	0.094489327	0.00083085	103797
0.4	0.104441218	0.000760383	129896
0.5	0.05434084	0.001238773	153317

TAG 54:1

thresh	pval	r2	nsnps
0.001	0.181893301	0.000397213	962
0.05	0.435798151	1.2584E-05	23182
0.1	0.141185541	0.000556513	41762
0.2	0.183296059	0.000392593	74775
0.3	0.107635405	0.000739428	103797
0.4	0.057405188	0.001197127	129896
0.5	0.105663283	0.000752277	153317

TAG 54:2

thresh	pval	r2	nsnps
0.001	0.033858641	0.001606369	962
0.05	0.386833144	3.98372E-05	23182
0.1	0.432972163	1.37271E-05	41762
0.2	0.256427518	0.000206259	74775
0.3	0.290003927	0.000147495	103797
0.4	0.486300385	5.68251E-07	129896
0.5	0.467772883	3.15022E-06	153317

TAG 54:3

thresh	pval	r2	nsnps
0.001	0.015652718	0.002230714	962
0.05	0.253324682	0.000212396	23182
0.1	0.232076252	0.000258089	41762
0.2	0.092144417	0.000848722	74775
0.3	0.126044506	0.000631635	103797
0.4	0.240455093	0.000239277	129896
0.5	0.211621362	0.000308861	153317

TAG 54:4

thresh	pval	r2	nsnps
0.001	0.06804351	0.001069589	962
0.05	0.013560129	0.002349422	23182
0.1	0.008968912	0.002694922	41762
0.2	0.001567335	0.004195115	74775
0.3	0.00253681	0.003775614	103797
0.4	0.00378431	0.003429945	129896
0.5	0.002765708	0.003700723	153317

TAG 54:5:PC1

thresh	pval	r2	nsnps
0.001	0.148504454	0.000523743	962
0.05	0.20335227	0.000331551	23182
0.1	0.05690568	0.001203747	41762
0.2	0.037663004	0.001522429	74775
0.3	0.066556907	0.001086023	103797
0.4	0.041436475	0.001447717	129896
0.5	0.064319156	0.00111155	153317

TAG 54:6:PC1

thresh	pval	r2	nsnps
0.001	0.052254426	0.001268632	962
0.05	0.431712175	1.42532E-05	23182
0.1	0.250266837	0.000218572	41762
0.2	0.113814751	0.000700916	74775
0.3	0.233564915	0.000254668	103797
0.4	0.169930923	0.000438705	129896
0.5	0.242105376	0.000235697	153317

TAG 54:7:PC1

thresh	pval	r2	nsnps
0.001	0.030980325	0.001676873	962
0.05	0.27373282	0.000174299	23182
0.1	0.4665862	3.38704E-06	41762
0.2	0.263856545	0.000192079	74775
0.3	0.423411003	1.7975E-05	103797
0.4	0.368269971	5.45222E-05	129896
0.5	0.458125513	5.3266E-06	153317

TAG 54:8

thresh	pval	r2	nsnps
0.001	0.240247645	0.00023973	962
0.05	0.398677092	3.17588E-05	23182
0.1	0.338066749	8.40576E-05	41762
0.2	0.217544087	0.000293407	74775
0.3	0.319771189	0.00010565	103797
0.4	0.267972758	0.000184524	129896
0.5	0.410123324	2.48708E-05	153317

TAG 54:9

thresh	pval	r2	nsnps
0.001	0.016992489	0.0021631	962
0.05	0.008576241	0.00273263	23182
0.1	0.020294231	0.002017766	41762
0.2	0.050710329	0.001291583	74775
0.3	0.03263128	0.00163562	103797
0.4	0.079215486	0.0009577	129896
0.5	0.059412912	0.001171149	153317

TAG 56:2

thresh	pval	r2	nsnps
0.001	0.277032714	0.00016862	962
0.05	0.191904455	0.000365283	23182
0.1	0.485074697	6.7453E-07	41762
0.2	0.456392657	5.77838E-06	74775
0.3	0.402677337	2.92502E-05	103797
0.4	0.261973098	0.000195606	129896
0.5	0.354252246	6.73269E-05	153317

TAG 56:3

thresh	pval	r2	nsnps
0.001	0.173296923	0.000426638	962
0.05	0.098125476	0.000804135	23182
0.1	0.119420308	0.000668093	41762
0.2	0.035133998	0.00157715	74775
0.3	0.039562796	0.001483855	103797
0.4	0.13008006	0.000610558	129896
0.5	0.103960255	0.000763604	153317

TAG 56:4

thresh	pval	r2	nsnps
0.001	0.139549025	0.000564136	962
0.05	0.004829878	0.00322052	23182
0.1	0.006617985	0.002951942	41762
0.2	0.003533536	0.003489006	74775
0.3	0.003719261	0.003444872	103797
0.4	0.013098415	0.002378179	129896
0.5	0.00934206	0.002660636	153317

TAG 56:5:PC1

thresh	pval	r2	nsnps
0.001	0.47431551	1.99938E-06	962
0.05	0.338435269	8.36526E-05	23182
0.1	0.394071041	3.47839E-05	41762
0.2	0.496425758	3.86662E-08	74775
0.3	0.458976225	5.11161E-06	103797
0.4	0.470800987	2.58503E-06	129896
0.5	0.455406988	6.04365E-06	153317

TAG 56:6:PC1

thresh	pval	r2	nsnps
0.001	0.450438484	7.4728E-06	962
0.05	0.342703984	7.90439E-05	23182
0.1	0.357384462	6.43333E-05	41762
0.2	0.460082507	4.83873E-06	74775
0.3	0.457820135	5.40486E-06	103797
0.4	0.470083327	2.7139E-06	129896
0.5	0.408995113	2.55103E-05	153317

TAG 56:7:PC1

thresh	pval	r2	nsnps
0.001	0.143916921	0.000544036	962
0.05	0.088070676	0.000881058	23182
0.1	0.117174971	0.000681012	41762
0.2	0.300151717	0.000132235	74775
0.3	0.104589427	0.000759394	103797
0.4	0.131750577	0.000602068	129896
0.5	0.109976413	0.000724535	153317

TAG 56:8:PC1

thresh	pval	r2	nsnps
0.001	0.153910303	0.00050084	962
0.05	0.174214922	0.000423401	23182
0.1	0.248504882	0.000222189	41762
0.2	0.416841288	2.12387E-05	74775
0.3	0.356369892	6.52945E-05	103797
0.4	0.427197384	1.62221E-05	129896
0.5	0.369610914	5.33761E-05	153317

TAG 56:9:PC1

thresh	pval	r2	nsnps
0.001	0.354255589	6.73236E-05	962
0.05	0.294399011	0.000140754	23182
0.1	0.215319941	0.000299135	41762
0.2	0.048963593	0.001318485	74775
0.3	0.10326407	0.000768297	103797
0.4	0.048866173	0.001320016	129896
0.5	0.057325427	0.00119818	153317

TAG 56:10

thresh	pval	r2	nsnps
0.001	0.148012656	0.00052588	962
0.05	0.10535846	0.000754289	23182
0.1	0.095412609	0.000823955	41762
0.2	0.12317422	0.000647138	74775
0.3	0.056020144	0.00121564	103797
0.4	0.148525734	0.000523651	129896
0.5	0.122034887	0.000653415	153317

TAG 58:6

thresh	pval	r2	nsnps
0.001	0.057772967	0.001192294	962
0.05	0.45909098	5.08295E-06	23182
0.1	0.237947345	0.000244795	41762
0.2	0.307118059	0.000122368	74775
0.3	0.210616651	0.000311547	103797
0.4	0.337959446	8.41758E-05	129896
0.5	0.260137166	0.000199089	153317

TAG 58:7

thresh	pval	r2	nsnps
0.001	0.345503188	7.61041E-05	962
0.05	0.128696705	0.000617691	23182
0.1	0.014154459	0.002313868	41762
0.2	0.009830649	0.00261782	74775
0.3	0.019247676	0.002060975	103797
0.4	0.018673468	0.002085741	129896
0.5	0.013388507	0.002359991	153317

TAG 58:8 PC1

thresh	pval	r2	nsnps
0.001	0.463807321	3.97546E-06	962
0.05	0.100562464	0.000786871	23182
0.1	0.032242139	0.00164514	41762
0.2	0.0941737	0.000833226	74775
0.3	0.017237408	0.002151343	103797
0.4	0.016547505	0.00218492	129896
0.5	0.015332248	0.002247782	153317

TAG 58:9 PC1

thresh	pval	r2	nsnps
0.001	0.2749919	0.000172117	962
0.05	0.230651713	0.000261397	23182
0.1	0.19812161	0.000346615	41762
0.2	0.449463121	7.77143E-06	74775
0.3	0.164382753	0.000459314	103797
0.4	0.168176748	0.000445123	129896
0.5	0.160310732	0.000475036	153317

TAG 58:10 PC1

thresh	pval	r2	nsnps
0.001	0.095012027	0.000826937	962
0.05	0.021554749	0.001968726	23182
0.1	0.079202004	0.000957824	41762
0.2	0.274263955	0.000173377	74775
0.3	0.189321554	0.000373294	103797
0.4	0.31720079	0.000108924	129896
0.5	0.276236788	0.000169978	153317

TAG 58:11 PC1

thresh	pval	r2	nsnps
0.001	0.357805632	6.39366E-05	962
0.05	0.087382699	0.00088669	23182
0.1	0.043578054	0.001408512	41762
0.2	0.090765383	0.00085948	74775
0.3	0.036080223	0.001556196	103797
0.4	0.101442453	0.000780757	129896
0.5	0.105197729	0.000755352	153317

TAG 58:12

thresh	pval	r2	nsnps
0.001	0.092708217	0.000844377	962
0.05	0.056163172	0.001213705	23182
0.1	0.017497674	0.002139039	41762
0.2	0.023073369	0.001913499	74775
0.3	0.016249065	0.002199901	103797
0.4	0.051054362	0.001286404	129896
0.5	0.04207479	0.001435807	153317

TAG 60:12 PC1

thresh	pval	r2	nsnps
0.001	0.29224319	0.000144035	962
0.05	0.158321798	0.000482908	23182
0.1	0.087878533	0.000882626	41762
0.2	0.256567639	0.000205985	74775
0.3	0.184518388	0.000388607	103797
0.4	0.239241171	0.000241936	129896
0.5	0.208309511	0.000317788	153317

Serum.TG (serum total triglycerides)

thresh	pval	r2	nsnps
0.001	0.439726388	1.06761E-06	1412
0.05	0.413270076	2.22895E-06	35088
0.1	0.087577202	8.53185E-05	63094
0.2	0.026289953	0.000174374	112128
0.3	0.020693245	0.000193071	155513
0.4	0.019993972	0.000195775	193882
0.5	0.023262038	0.000183901	228369

PC aa - Draisma et al., 2015

PC 24:0

thresh	pval	r2	nsnps
0.001	0.113076419	0.000383307	900
0.05	0.081042123	0.00051143	21379
0.1	0.005668297	0.001676629	38383
0.2	0.007980098	0.001518671	68472
0.3	0.005808242	0.001665318	94926
0.4	0.005627103	0.001680013	118543
0.5	0.007762254	0.001531395	139633

PC 28:1

thresh	pval	r2	nsnps
0.001	0.00074349	0.001349006	938
0.05	0.058745432	0.000327635	22493
0.1	0.020835679	0.000554607	40450
0.2	0.005974143	0.000844592	72166
0.3	0.003187751	0.000994555	99996
0.4	0.001963092	0.001111663	124985
0.5	0.004275382	0.000924196	147284

PC 30:0

thresh	pval	r2	nsnps
0.001	0.160494446	0.000131717	939
0.05	0.241980165	6.55223E-05	22488
0.1	0.168127753	0.000123659	40448
0.2	0.165347922	0.000126536	72195
0.3	0.309792362	3.29608E-05	100017
0.4	0.323953336	2.78917E-05	124995
0.5	0.229486098	7.33422E-05	147290

PC 32:0

thresh	pval	r2	nsnps
0.001	0.061722496	0.000317331	939
0.05	0.370532692	1.46037E-05	22491
0.1	0.095795142	0.000228053	40443
0.2	0.057364944	0.000332676	72174
0.3	0.025648368	0.000507903	100003
0.4	0.017719106	0.000591498	124961
0.5	0.019650894	0.000567951	147257

PC 32:1

thresh	pval	r2	nsnps
0.001	0.39796862	8.94571E-06	941
0.05	0.152094319	0.000141207	22492
0.1	0.214910104	8.33712E-05	40446
0.2	0.076943295	0.000271939	72186
0.3	0.019676804	0.000567728	100027
0.4	0.017255826	0.000597624	124996
0.5	0.028684485	0.00048301	147284

PC 32:2

thresh	pval	r2	nsnps
0.001	0.431792269	3.94753E-06	941
0.05	0.493332541	3.73556E-08	22507
0.1	0.342930207	2.18777E-05	40452
0.2	0.480303496	3.26231E-07	72193
0.3	0.354704422	1.85701E-05	100043
0.4	0.446195517	2.44724E-06	125036
0.5	0.389915528	1.04496E-05	147317

PC 32:3

thresh	pval	r2	nsnps
0.001	0.001372077	0.001198829	940
0.05	0.012547848	0.000670712	22490
0.1	0.003199206	0.000993693	40446
0.2	0.000860738	0.001313003	72178
0.3	7.4663E-05	0.001921218	100014
0.4	1.84925E-05	0.002273443	124986
0.5	9.60549E-05	0.001857957	147266

PC 34:1

thresh	pval	r2	nsnps
0.001	0.293549535	3.94398E-05	941
0.05	0.383687108	1.17031E-05	22483
0.1	0.220503806	7.93949E-05	40445
0.2	0.02579529	0.000506625	72183
0.3	0.009253022	0.00074168	100010
0.4	0.006020607	0.000842869	124989
0.5	0.009394356	0.000738135	147286

PC 34:2

thresh	pval	r2	nsnps
0.001	0.007489749	0.000791192	939
0.05	0.103380618	0.000213131	22493
0.1	0.012399713	0.000673461	40448
0.2	0.003198063	0.000993779	72171
0.3	0.000814402	0.0013266	100012
0.4	0.000756048	0.001344885	124985
0.5	0.001787153	0.001134464	147282

PC 34:3

thresh	pval	r2	nsnps
0.001	0.4138743	6.33127E-06	940
0.05	0.223528224	7.73043E-05	22486
0.1	0.161235471	0.000130895	40434
0.2	0.054252307	0.0003444	72182
0.3	0.02633442	0.000501933	100021
0.4	0.012979835	0.000662886	125001
0.5	0.023139699	0.000530936	147308

PC 34:4

thresh	pval	r2	nsnps
0.001	0.226877973	7.5051E-05	942
0.05	0.461942682	1.22065E-06	22497
0.1	0.469806484	7.67454E-07	40442
0.2	0.451556794	1.98154E-06	72194
0.3	0.324333188	2.77591E-05	100038
0.4	0.261289609	5.46655E-05	125030
0.5	0.394573412	9.56304E-06	147303

PC 36:0

thresh	pval	r2	nsnps
0.001	0.429123907	4.26581E-06	939
0.05	0.015461	0.000622605	22487
0.1	0.060006246	0.000323184	40429
0.2	0.042612768	0.00039606	72165
0.3	0.03433885	0.000443114	100011
0.4	0.035743728	0.000434318	124995
0.5	0.024973657	0.000513806	147271

PC 36:1

thresh	pval	r2	nsnps
0.001	0.165735149	0.000126165	941
0.05	0.458115893	1.48002E-06	22482
0.1	0.434734161	3.61279E-06	40445
0.2	0.46442424	1.06667E-06	72184
0.3	0.343898709	2.16025E-05	100009
0.4	0.317410593	3.01774E-05	124992
0.5	0.41679069	5.90586E-06	147280

PC 36:2

thresh	pval	r2	nsnps
0.001	0.002151654	0.001089428	940
0.05	0.136560337	0.000160592	22486
0.1	0.077753114	0.00026974	40443
0.2	0.068710893	0.000295035	72174
0.3	0.025970414	0.000505044	100031
0.4	0.022800357	0.000534263	125024
0.5	0.044891682	0.000384822	147319

PC 36:3

thresh	pval	r2	nsnps
0.001	0.022394858	0.000538378	939
0.05	0.288395825	4.16536E-05	22485
0.1	0.134185145	0.00016383	40443
0.2	0.175877536	0.000115964	72169
0.3	0.057943096	0.000330567	100000
0.4	0.048946344	0.000366329	124982
0.5	0.085253445	0.000251203	147268

PC 36:4

thresh	pval	r2	nsnps
0.001	0.05351372	0.000347302	939
0.05	0.053392337	0.000347783	22492
0.1	0.220825178	7.91612E-05	40446
0.2	0.180341659	0.000111723	72179
0.3	0.16796828	0.000123806	100024
0.4	0.278586571	4.60838E-05	125005
0.5	0.218505031	8.07831E-05	147286

PC 36:5

thresh	pval	r2	nsnps
0.001	0.323415219	2.80863E-05	938
0.05	0.49117651	6.54604E-08	22492
0.1	0.393556432	9.75822E-06	40465
0.2	0.249454588	6.11777E-05	72205
0.3	0.304818002	3.4881E-05	100025
0.4	0.384548465	1.15299E-05	125000
0.5	0.452766827	1.88433E-06	147309

PC 36:6

thresh	pval	r2	nsnps
0.001	0.272993655	4.87622E-05	940
0.05	0.245356476	6.35296E-05	22486
0.1	0.32321636	2.81466E-05	40461
0.2	0.337487818	2.35199E-05	72215
0.3	0.319666987	2.93755E-05	100035
0.4	0.25727043	5.68231E-05	125016
0.5	0.181291885	0.000110872	147306

PC 38:0

thresh	pval	r2	nsnps
0.001	0.336756354	2.37388E-05	941
0.05	0.4180173	5.72824E-06	22497
0.1	0.384974129	1.14365E-05	40436
0.2	0.490412734	1.72445E-08	72169
0.3	0.403655691	7.95467E-06	100013
0.4	0.435545602	3.52131E-06	125012
0.5	0.497107586	7.02947E-09	147294

PC 38:1

thresh	pval	r2	nsnps
0.001	0.467593719	4.41746E-06	834
0.05	0.273510104	0.000242244	19726
0.1	0.43537637	1.76833E-05	35355
0.2	0.337393316	0.000117598	62873
0.3	0.336559502	0.000118881	87055
0.4	0.42062052	2.68017E-05	108410
0.5	0.37180836	7.14559E-05	127585

PC 38:2

thresh	pval	r2	nsnps
0.001	0.029338601	0.001720284	962
0.05	0.001454948	0.004260226	23182
0.1	0.011862444	0.002460663	41762
0.2	0.002353457	0.003840747	74775
0.3	0.000376163	0.00545487	103797

PC 38:3

thresh	pval	r2	nsnps
0.001	0.0092093	0.000742788	937
0.05	0.499898907	8.58813E-12	22483
0.1	0.371917991	1.42815E-05	40448
0.2	0.159037575	0.000133313	72172
0.3	0.429427691	4.2295E-06	100006

PC 38:4

thresh	pval	r2	nsnps
0.001	0.006558335	0.000822736	938
0.05	0.144052533	0.000150932	22491
0.1	0.237255509	6.84102E-05	40445
0.2	0.112191753	0.000197428	72164
0.3	0.129427427	0.000170514	99991

PC 38:5

thresh	pval	r2	nsnps
0.001	0.248757718	6.15429E-05	938
0.05	0.036929771	0.000427175	22489
0.1	0.038262246	0.000419438	40457
0.2	0.021242972	0.000550228	72171
0.3	0.012924283	0.000663877	100005

0.4	0.000668416	0.004944846	129896
0.5	5.29272E-05	0.00721341	153317

0.4	0.352108809	1.92751E-05	124980
0.5	0.236943752	6.85943E-05	147274

0.4	0.212025921	8.54777E-05	124976
0.5	0.148203562	0.000145828	147246

0.4	0.017523243	0.000593954	124987
0.5	0.009192934	0.000743105	147263

PC 38:6

thresh	pval	r2	nsnps
0.001	0.442188379	2.82839E-06	939
0.05	0.279589088	4.56228E-05	22479
0.1	0.244083085	6.42705E-05	40450
0.2	0.273184342	4.86631E-05	72180
0.3	0.217755434	8.13237E-05	99988
0.4	0.105617936	0.000209011	124961
0.5	0.088591141	0.000243531	147260

PC 40:1

thresh	pval	r2	nsnps
0.001	0.221765241	0.000121855	938
0.05	0.025235289	0.00079374	22246
0.1	0.043150482	0.000610451	39993
0.2	0.006049549	0.001305973	71377
0.3	0.006443383	0.001282819	98911
0.4	0.005284848	0.001355715	123605
0.5	0.004030817	0.001455919	145655

PC 40:2

thresh	pval	r2	nsnps
0.001	0.026257174	0.000502858	936
0.05	0.342355196	2.20591E-05	22479
0.1	0.355955555	1.82465E-05	40440
0.2	0.330684696	2.56706E-05	72194
0.3	0.206436478	8.97087E-05	100029
0.4	0.20074593	9.41692E-05	125031
0.5	0.179758563	0.000112327	147310

PC 40:3

thresh	pval	r2	nsnps
0.001	0.018471088	0.00058226	940
0.05	0.221990384	7.83984E-05	22485
0.1	0.09908487	0.000221518	40447
0.2	0.266713154	5.18933E-05	72177
0.3	0.230727125	7.25643E-05	100005
0.4	0.186448208	0.000106228	124989
0.5	0.207231004	8.91002E-05	147290

PC 40:4

thresh	pval	r2	nsnps
0.001	0.027404639	0.000493306	938
0.05	0.223648358	7.72639E-05	22491
0.1	0.243562037	6.46049E-05	40439
0.2	0.064391911	0.000308636	72154
0.3	0.060766892	0.00032072	99987
0.4	0.111649416	0.000198407	124984
0.5	0.045038224	0.000384326	147260

PC 40:5

thresh	pval	r2	nsnps
0.001	0.008382699	0.00076513	936
0.05	0.39913014	8.74106E-06	22486
0.1	0.172949222	0.000118865	40454
0.2	0.126959192	0.000174137	72174
0.3	0.118183409	0.000187584	100000
0.4	0.12174307	0.000181987	124945
0.5	0.06627966	0.00030264	147212

PC 40:6

thresh	pval	r2	nsnps
0.001	0.126095249	0.000175363	937
0.05	0.281523881	4.47361E-05	22488
0.1	0.458127452	1.479E-06	40452
0.2	0.419531353	5.51718E-06	72186
0.3	0.482924909	2.4519E-07	100004
0.4	0.308110226	3.36024E-05	124961
0.5	0.259710082	5.55154E-05	147255

PC 42:0

thresh	pval	r2	nsnps
0.001	0.136004429	0.000161339	939
0.05	0.023738455	0.000525191	22504
0.1	0.002934041	0.001014512	40444
0.2	0.008549817	0.000760091	72165
0.3	0.001871936	0.001123205	99990
0.4	0.001775535	0.001136049	124993
0.5	0.003925916	0.000944587	147269

PC 42:1

thresh	pval	r2	nsnps
0.001	0.083274969	0.000255881	939
0.05	0.057891366	0.00033071	22488
0.1	0.005166795	0.000879055	40434
0.2	0.052523963	0.00035126	72164
0.3	0.029998806	0.000472936	100004
0.4	0.012315075	0.000675047	124994
0.5	0.029833597	0.00047416	147286

PC 42:2

thresh	pval	r2	nsnps
0.001	0.124605648	0.000177629	940
0.05	0.20513634	9.07118E-05	22487
0.1	0.065360181	0.000305537	40431
0.2	0.186836317	0.000105883	72180
0.3	0.169527611	0.000122283	100000
0.4	0.190315211	0.00010284	125003
0.5	0.248335661	6.1818E-05	147280

PC 42:4

thresh	pval	r2	nsnps
0.001	0.070609414	0.000289424	940
0.05	0.215165066	8.31648E-05	22486
0.1	0.305584381	3.45643E-05	40445
0.2	0.103789583	0.000212364	72176
0.3	0.060148521	0.000322688	100006
0.4	0.054974268	0.000341605	124978
0.5	0.03171147	0.00046065	147253

PC 42:5

thresh	pval	r2	nsnps
0.001	0.079552039	0.000265212	939
0.05	0.074890463	0.000277484	22488
0.1	0.082212355	0.000258569	40428
0.2	0.146666509	0.000147716	72170
0.3	0.205697746	9.02654E-05	100012
0.4	0.229853893	7.31219E-05	124999
0.5	0.292600854	3.98521E-05	147281

PC 42:6

thresh	pval	r2	nsnps
0.001	0.208599346	9.97061E-05	935
0.05	0.050404302	0.00040784	22441
0.1	0.021231722	0.000623444	40347
0.2	0.048381478	0.000417744	71990
0.3	0.048004059	0.000419641	99742
0.4	0.052488144	0.000398083	124702
0.5	0.079351466	0.0003009	146936

PC ae - Draisma et al., 2015

PC 30:0

thresh	pval	r2	nsnps
0.001	0.129656672	0.000192626	940
0.05	0.280827054	5.09977E-05	22455
0.1	0.113814191	0.000220357	40370
0.2	0.036232407	0.000488342	72035
0.3	0.01029508	0.000811352	99777
0.4	0.008809472	0.000852573	124740
0.5	0.019460602	0.000645409	147001

PC 32:1

thresh	pval	r2	nsnps
0.001	0.0127323	0.000667337	939
0.05	0.347041997	2.06871E-05	22484
0.1	0.101147887	0.000217377	40432
0.2	0.016160858	0.000612456	72159
0.3	0.001906695	0.001118738	99997
0.4	0.000737665	0.001350942	124979
0.5	0.001193129	0.001232968	147278

PC 32:2

thresh	pval	r2	nsnps
0.001	0.010422929	0.000713791	938
0.05	0.035362096	0.00043667	22496
0.1	0.001398094	0.001194246	40448
0.2	0.000191843	0.001684861	72182
0.3	1.65469E-05	0.002301613	100028
0.4	1.51522E-06	0.002910598	125007
0.5	2.68364E-06	0.002764497	147310

PC 34:0

thresh	pval	r2	nsnps
0.001	0.084497475	0.000252955	940
0.05	0.395162185	9.45398E-06	22497
0.1	0.468895561	8.14555E-07	40461
0.2	0.310773421	3.25883E-05	72196
0.3	0.183151328	0.000109137	100015
0.4	0.174498575	0.000117284	125023
0.5	0.28198759	4.4512E-05	147305

PC 34:1

thresh	pval	r2	nsnps
0.001	0.19575563	9.81954E-05	940
0.05	0.215371716	8.30269E-05	22482

PC 34:2

thresh	pval	r2	nsnps
0.001	0.159234358	0.000133078	941
0.05	0.214386155	8.37283E-05	22487

PC 34:3

thresh	pval	r2	nsnps
0.001	0.113480743	0.000195194	939
0.05	0.491526479	6.03375E-08	22478

PC 36:0

thresh	pval	r2	nsnps
0.001	0.179697994	0.000112354	939
0.05	0.002451386	0.001058155	22491

0.1	0.415158503	6.1418E-06	40435
0.2	0.362179136	1.66315E-05	72186
0.3	0.162980353	0.000129038	100008
0.4	0.113062495	0.000195924	125013
0.5	0.166537323	0.000125297	147303

0.1	0.330169402	2.58237E-05	40444
0.2	0.214746775	8.3467E-05	72186
0.3	0.142581532	0.000152745	100015
0.4	0.12408917	0.000178311	125006
0.5	0.172553026	0.000119192	147308

0.1	0.165605215	0.00012625	40451
0.2	0.073945917	0.000279964	72197
0.3	0.066869708	0.000300644	100021
0.4	0.046068311	0.00037926	125010
0.5	0.047989616	0.0003705	147302

0.1	0.014782848	0.000633075	40436
0.2	0.007091621	0.000804277	72156
0.3	0.014478003	0.000637869	99991
0.4	0.010682503	0.000708252	124992
0.5	0.005915993	0.000847136	147281

PC 36:1

thresh	pval	r2	nsnps
0.001	0.043764357	0.000390354	939
0.05	0.101936358	0.000215894	22481
0.1	0.169052356	0.000122716	40431
0.2	0.2083581	8.82076E-05	72178
0.3	0.234953951	6.98374E-05	100017
0.4	0.334634058	2.44025E-05	125023
0.5	0.22077413	7.92072E-05	147308

PC 36:2

thresh	pval	r2	nsnps
0.001	0.031533496	0.000461955	940
0.05	0.403693604	7.94935E-06	22491
0.1	0.283039613	4.4039E-05	40456
0.2	0.260320701	5.51841E-05	72185
0.3	0.160386348	0.000131835	100037
0.4	0.111176574	0.00019914	125034
0.5	0.208951304	8.77594E-05	147344

PC 36:3

thresh	pval	r2	nsnps
0.001	0.314027787	3.13858E-05	940
0.05	0.038753942	0.000416656	22496
0.1	0.221524574	7.86774E-05	40459
0.2	0.23831326	6.77394E-05	72198
0.3	0.39205209	1.00377E-05	100043
0.4	0.396589165	9.19243E-06	125024
0.5	0.322461339	2.83978E-05	147328

PC 36:4

thresh	pval	r2	nsnps
0.001	0.109425215	0.000202185	938
0.05	0.027393223	0.000493201	22491
0.1	0.232056795	7.1678E-05	40437
0.2	0.22924022	7.3503E-05	72188
0.3	0.274023764	4.8257E-05	100028
0.4	0.466389822	9.51532E-07	124987
0.5	0.367817916	1.52466E-05	147270

PC 36:5

thresh	pval	r2	nsnps
0.001	0.076699771	0.000272584	940
0.05	0.280109177	4.53877E-05	22497
0.1	0.34560621	2.11041E-05	40458
0.2	0.246630653	6.27862E-05	72219
0.3	0.19025223	0.000102867	100049
0.4	0.087911671	0.000245098	125035
0.5	0.121280719	0.000182654	147317

PC 38:0

thresh	pval	r2	nsnps
0.001	0.362065238	1.66624E-05	940
0.05	0.499117349	6.5477E-10	22491
0.1	0.374742394	1.36388E-05	40458
0.2	0.364601856	1.60304E-05	72201
0.3	0.405285767	7.68466E-06	100014
0.4	0.475856398	4.90507E-07	124991
0.5	0.414182612	6.28699E-06	147284

PC 38:1

thresh	pval	r2	nsnps
0.001	0.167508329	0.000142145	940
0.05	0.000953602	0.001472877	22429
0.1	0.003410301	0.001118923	40328
0.2	7.9077E-05	0.002180634	72000
0.3	1.75135E-05	0.00261564	99770
0.4	1.59283E-05	0.002643135	124701
0.5	5.14217E-06	0.002971695	146937

PC 38:2

thresh	pval	r2	nsnps
0.001	0.058205344	0.00032953	941
0.05	0.064807263	0.000307095	22496
0.1	0.075446746	0.000275827	40451
0.2	0.021200366	0.000550609	72195
0.3	0.04437854	0.000387246	100024
0.4	0.075256938	0.00027634	125020
0.5	0.035508672	0.000435705	147330

PC 38:3

thresh	pval	r2	nsnps
0.001	0.00577929	0.000852566	939
0.05	0.133650808	0.000164563	22496
0.1	0.285482304	4.29403E-05	40453
0.2	0.147957606	0.000146106	72185
0.3	0.2532128	5.90387E-05	100007
0.4	0.326991792	2.68707E-05	125010
0.5	0.19900897	9.55292E-05	147303

PC 38:4

thresh	pval	r2	nsnps
0.001	0.067854603	0.000297622	938
0.05	0.000551787	0.001422522	22486
0.1	0.004520208	0.000910901	40438
0.2	0.000615954	0.00139537	72161
0.3	0.000635615	0.001387621	100004
0.4	0.001902208	0.00111931	124988
0.5	0.000327062	0.001552054	147274

PC 38:5

thresh	pval	r2	nsnps
0.001	0.232532778	7.13636E-05	940
0.05	0.008741007	0.000754907	22482
0.1	0.093149235	0.000233544	40436
0.2	0.054461793	0.000343585	72169
0.3	0.07758377	0.000270183	100010
0.4	0.169525816	0.00012222	125002
0.5	0.128249847	0.000172164	147282

PC 38:6

thresh	pval	r2	nsnps
0.001	0.447828909	2.30038E-06	939
0.05	0.122119376	0.000181334	22497
0.1	0.239840748	6.68143E-05	40454
0.2	0.322740725	2.83057E-05	72190
0.3	0.409793258	6.95718E-06	100026
0.4	0.418315636	5.68679E-06	125016
0.5	0.387019222	1.10237E-05	147324

PC 40:1

thresh	pval	r2	nsnps
0.001	0.244868591	6.38075E-05	938
0.05	0.152903918	0.000140246	22481
0.1	0.356704266	1.80413E-05	40443
0.2	0.159130407	0.00013321	72189
0.3	0.136104329	0.000161226	100019
0.4	0.248268119	6.18319E-05	125014
0.5	0.187825731	0.000104968	147286

PC 40:2

thresh	pval	r2	nsnps
0.001	0.021333769	0.000549264	941
0.05	0.438641961	3.18847E-06	22492
0.1	0.375177828	1.35373E-05	40437
0.2	0.493451562	3.60337E-08	72180
0.3	0.470141589	7.50481E-07	100021
0.4	0.473847467	5.75499E-07	125035
0.5	0.397608397	9.00802E-06	147325

PC 40:3

thresh	pval	r2	nsnps
0.001	0.008153214	0.000771236	940
0.05	0.145796083	0.000148726	22491
0.1	0.26747964	5.1478E-05	40448
0.2	0.103788687	0.000212366	72186
0.3	0.181116256	0.000111005	100018
0.4	0.216925408	8.19026E-05	125005
0.5	0.135010029	0.000162683	147298

PC 40:4

thresh	pval	r2	nsnps
0.001	0.007161835	0.000801739	939
0.05	0.178522042	0.000113428	22487
0.1	0.443956495	2.65654E-06	40430
0.2	0.20698213	8.92427E-05	72160
0.3	0.292932159	3.96956E-05	99990
0.4	0.487041237	1.41148E-07	124975
0.5	0.276210128	4.72039E-05	147266

PC 40:5

thresh	pval	r2	nsnps
0.001	0.048615507	0.000367729	940
0.05	0.310820364	3.25708E-05	22482
0.1	0.393545139	9.75512E-06	40451
0.2	0.35109454	1.95511E-05	72175
0.3	0.386227201	1.11817E-05	100022
0.4	0.497866766	3.82363E-09	124988
0.5	0.356719286	1.8035E-05	147295

PC 40:6

thresh	pval	r2	nsnps
0.001	0.468498726	8.35516E-07	940
0.05	0.042559876	0.000396328	22489
0.1	0.019229025	0.000572803	40459
0.2	0.010144509	0.000720101	72184
0.3	0.008707414	0.000755809	100025
0.4	0.00441235	0.000916665	125017
0.5	0.001836759	0.001127812	147307

PC 42:0

thresh	pval	r2	nsnps
0.001	0.271091478	5.6245E-05	941
0.05	0.14952671	0.000163259	22444
0.1	0.128798814	0.000194019	40336
0.2	0.283861614	4.94321E-05	71982
0.3	0.358942911	1.97617E-05	99746
0.4	0.408201301	8.1612E-06	124678
0.5	0.406506781	8.47101E-06	146936

PC 42:1

thresh	pval	r2	nsnps
0.001	0.015357861	0.000624224	937
0.05	0.463069137	1.14941E-06	22491
0.1	0.178326145	0.000113628	40449
0.2	0.349869482	1.98933E-05	72181
0.3	0.304366449	3.50434E-05	99997
0.4	0.43655228	3.4117E-06	124991
0.5	0.325056094	2.75185E-05	147294

PC 42:2

thresh	pval	r2	nsnps
0.001	0.048032238	0.00037036	939

PC 42:3

thresh	pval	r2	nsnps
0.001	0.02069401	0.000556151	938

PC 42:4

thresh	pval	r2	nsnps
0.001	0.011211545	0.000696822	935

PC 42:5

thresh	pval	r2	nsnps
0.001	0.114050896	0.000219909	938

0.05	0.024981823	0.000513801	22490
0.1	0.028812823	0.000481954	40450
0.2	0.004707163	0.000901357	72191
0.3	0.003951567	0.000943155	100034
0.4	0.004266895	0.000924794	125033
0.5	0.001173776	0.001237132	147332

0.05	0.160486801	0.000131708	22491
0.1	0.051201187	0.000356679	40446
0.2	0.118303673	0.000187291	72180
0.3	0.14210286	0.000153354	100014
0.4	0.145988193	0.000148489	124997
0.5	0.218051074	8.11036E-05	147301

0.05	0.105983809	0.000208314	22487
0.1	0.032017866	0.000458527	40450
0.2	0.09020377	0.00023991	72175
0.3	0.057108428	0.000333574	100018
0.4	0.036475177	0.000429883	125009
0.5	0.083108251	0.000256284	147301

0.05	0.08330131	0.000289625	22440
0.1	0.056903935	0.00037851	40342
0.2	0.104003793	0.000239977	72007
0.3	0.043450458	0.000443636	99777
0.4	0.030438975	0.000531774	124702
0.5	0.074531427	0.000315138	146964

PC 44:3

thresh	pval	r2	nsnps
0.001	0.004708009	0.000901074	938
0.05	0.185310641	0.000107172	22489
0.1	0.064820566	0.000307052	40447
0.2	0.320406274	2.91052E-05	72176
0.3	0.41006487	6.91277E-06	100009
0.4	0.419280394	5.5499E-06	125013
0.5	0.465023602	1.03039E-06	147299

PC 44:4

thresh	pval	r2	nsnps
0.001	0.023002197	0.000532349	939
0.05	0.261093619	5.4776E-05	22492
0.1	0.141123267	0.000154628	40442
0.2	0.200392642	9.44143E-05	72189
0.3	0.139942794	0.000156153	100024
0.4	0.146543217	0.000147828	125025
0.5	0.24089276	6.61766E-05	147314

PC 44:5

thresh	pval	r2	nsnps
0.001	0.039057889	0.000414955	938
0.05	0.043957498	0.000389352	22489
0.1	0.005706949	0.000855439	40435
0.2	0.003720405	0.000957465	72169
0.3	0.001073605	0.0012588	99990
0.4	0.000706621	0.001361527	124973
0.5	0.002401964	0.001062796	147278

PC 44:6

thresh	pval	r2	nsnps
0.001	0.028306059	0.0004859	937
0.05	0.001231382	0.001225416	22502
0.1	9.73439E-05	0.001854861	40449
0.2	6.15703E-05	0.001969967	72176
0.3	1.94845E-05	0.002260508	100019
0.4	8.186E-06	0.002480624	124993
0.5	4.59926E-05	0.002043446	147289

LPC - Rhee et al., 2013 & Draisma et al., 2015

LPC 14:0

thresh	pval	r2	nsnps
0.001	0.238769776	0.000242975	962
0.05	0.298334172	0.00013489	23182
0.1	0.377287376	4.70735E-05	41762
0.2	0.345801787	7.57943E-05	74775
0.3	0.294427486	0.000140711	103797
0.4	0.123373894	0.000646046	129896
0.5	0.166135468	0.000452704	153317

LPC 16:0

thresh	pval	r2	nsnps
0.001	0.209914336	8.70241E-05	939
0.05	0.000154265	0.001739309	22496
0.1	1.11612E-05	0.002401515	40461
0.2	3.99285E-06	0.002663108	72181
0.3	8.81846E-07	0.003049188	100001
0.4	1.43909E-06	0.002923786	124994
0.5	1.13586E-06	0.002984352	147288

LPC 16:1

thresh	pval	r2	nsnps
0.001	0.058624106	0.0003282	939
0.05	0.07353265	0.000281222	22485
0.1	0.030601571	0.000468717	40442
0.2	0.016601211	0.000606547	72181
0.3	0.044878021	0.000385042	100015
0.4	0.068391732	0.000296114	124996
0.5	0.062820748	0.000313736	147303

LPC 17:0

thresh	pval	r2	nsnps
0.001	0.131898366	0.000166972	939
0.05	3.43798E-06	0.002701271	22495
0.1	1.10379E-07	0.003583062	40451
0.2	1.02267E-09	0.004793237	72191
0.3	3.76738E-10	0.005052321	100026
0.4	4.23729E-10	0.005021812	125019
0.5	8.52107E-11	0.005438406	147292

LPC 18:0

thresh	pval	r2	nsnps
0.001	0.460783516	1.29638E-06	939
0.05	1.81113E-08	0.004049261	22486
0.1	1.92021E-12	0.006425419	40452
0.2	8.99712E-17	0.009027076	72184
0.3	5.83191E-18	0.009742117	100026
0.4	1.5903E-18	0.010081759	124995
0.5	1.94595E-19	0.010630906	147278

LPC 18:1

thresh	pval	r2	nsnps
0.001	0.050135525	0.000361206	940
0.05	0.013953051	0.000646288	22511
0.1	0.003725792	0.000957246	40475
0.2	0.001686825	0.001148664	72213
0.3	0.001409808	0.00119237	100056
0.4	0.004805256	0.000896442	125044
0.5	0.005286943	0.000873707	147338

LPC 18:2

thresh	pval	r2	nsnps
0.001	0.32773726	2.66272E-05	938
0.05	0.209268318	8.75324E-05	22490
0.1	0.155146928	0.000137688	40453
0.2	0.070118506	0.000290936	72197
0.3	0.085545571	0.000250551	100016
0.4	0.116862404	0.000189662	125015
0.5	0.100111483	0.000219445	147266

LPC 20:3

thresh	pval	r2	nsnps
0.001	0.189335796	0.000103633	938
0.05	0.01291935	0.000663965	22492
0.1	0.001148713	0.001242247	40460
0.2	0.000105782	0.001833765	72185
0.3	0.000390751	0.00150791	100018
0.4	0.000377057	0.001516754	125010
0.5	0.0001577	0.001733806	147289

LPC 20:4

thresh	pval	r2	nsnps
0.001	0.484784939	1.9463E-07	940
0.05	0.006705375	0.000817387	22500
0.1	0.009919983	0.000725418	40459
0.2	0.002206153	0.001083515	72186
0.3	0.000961398	0.001286027	100008
0.4	0.004911593	0.000891228	124992
0.5	0.003458232	0.000975121	147256

LPC 20:5 PC1

thresh	pval	r2	nsnps
0.001	0.026263996	0.001808994	962
0.05	5.12351E-05	0.007242739	23182
0.1	0.00035468	0.005507207	41762
0.2	0.003528086	0.003490337	74775
0.3	0.000292919	0.005677678	103797
0.4	0.000618215	0.005013928	129896
0.5	0.000757788	0.004833935	153317

LPC 22:6 PC1

thresh	pval	r2	nsnps
0.001	0.158878958	0.00048069	962
0.05	0.00423375	0.003333463	23182
0.1	0.048326772	0.001328554	41762
0.2	0.060768252	0.001154154	74775
0.3	0.018034339	0.002114262	103797
0.4	0.014687082	0.0022833	129896
0.5	0.036149374	0.001554688	153317

LPC 28:0

thresh	pval	r2	nsnps
0.001	0.284390737	4.91847E-05	938
0.05	0.155309808	0.000155704	22447
0.1	0.357885208	2.00818E-05	40357
0.2	0.366858429	1.75288E-05	72004
0.3	0.272680529	5.53898E-05	99772
0.4	0.287797913	4.74707E-05	124724
0.5	0.344314931	2.43215E-05	146989

LPC 28:1

thresh	pval	r2	nsnps
0.001	0.005282401	0.000873911	938
0.05	0.414346795	6.26174E-06	22493
0.1	0.292948028	3.96942E-05	40451
0.2	0.385095483	1.14132E-05	72179
0.3	0.460550498	1.31206E-06	100018
0.4	0.419062232	5.58186E-06	125018
0.5	0.460734319	1.29982E-06	147309

LPC 20:2

thresh	pval	r2	nsnps
0.001	0.026897004	0.000571475	868
0.05	0.058379873	0.00037808	20363
0.1	0.020539028	0.00064117	36488
0.2	0.017538695	0.00068243	64867
0.3	0.030706813	0.000537635	89563
0.4	0.04057416	0.000467421	111600
0.5	0.047192241	0.000429966	131229

LPE - Rhee et al., 2013 & Shin et al., 2014

LPE 16:0

thresh	pval	r2	nsnps
0.001	0.318469114	0.000107301	962
0.05	0.025640249	0.001828331	23182
0.1	0.016456319	0.002189468	41762
0.2	0.001366068	0.00431544	74775
0.3	0.000656813	0.004960336	103797
0.4	0.00227535	0.003870077	129896
0.5	0.001459737	0.004257349	153317

LPE 18:0

thresh	pval	r2	nsnps
0.001	0.34818905	7.33437E-05	962
0.05	0.028872179	0.001733089	23182
0.1	0.035248905	0.001574574	41762
0.2	0.004596576	0.003262927	74775
0.3	0.003639814	0.00346347	103797
0.4	0.00710516	0.002891681	129896
0.5	0.007224315	0.002877588	153317

LPE 18:1

thresh	pval	r2	nsnps
0.001	0.420374023	1.94485E-05	962
0.05	0.189151606	0.000373826	23182
0.1	0.304058052	0.000126643	41762
0.2	0.168099688	0.000445407	74775
0.3	0.176996963	0.000413733	103797
0.4	0.261838627	0.00019586	129896
0.5	0.224275131	0.000276606	153317

LPE 18:2

thresh	pval	r2	nsnps
0.001	0.02067162	0.002002756	962
0.05	0.002071589	0.003951697	23182
0.1	7.25065E-05	0.006929578	41762
0.2	0.003132216	0.003593057	74775
0.3	0.001839892	0.004055069	103797
0.4	0.001888796	0.004032189	129896
0.5	0.003730572	0.003442258	153317

LPE 20:4

thresh	pval	r2	nsnps
0.001	0.325739908	9.82801E-05	962
0.05	0.165982038	0.000453279	23182
0.1	0.275718352	0.000170867	41762
0.2	0.135631966	0.000582848	74775
0.3	0.0615851	0.001144113	103797
0.4	0.092661431	0.000844736	129896
0.5	0.093683114	0.000836936	153317

LPE 22:6

thresh	pval	r2	nsnps
0.001	0.207332598	0.000320462	962
0.05	0.000725498	0.004872403	23182
0.1	0.002096041	0.003941481	41762
0.2	0.000308722	0.005630829	74775
0.3	4.12636E-05	0.007438238	103797
0.4	8.39534E-05	0.006797576	129896
0.5	2.61986E-05	0.007849254	153317

LPI - Shin et al., 2014

LPI 16:0

thresh	pval	r2	nsnps
0.001	0.100191696	0.000274185	867
0.05	0.327288798	3.35E-05	20365
0.1	0.176728202	0.000143995	36484
0.2	0.090787696	0.000298447	64861
0.3	0.114877115	0.000241212	89586
0.4	0.113075661	0.000244978	111594
0.5	0.163604835	0.000160534	131228

LPI 18:0

thresh	pval	r2	nsnps
0.001	0.18606102	0.00010985	869
0.05	0.319790112	3.02E-05	20368
0.1	0.062248124	0.000325395	36488
0.2	0.030429795	0.000484478	64867
0.3	0.027792679	0.000505242	89587
0.4	0.048631813	0.000379165	111588
0.5	0.094384145	0.000238174	131215

LPI 20:4

thresh	pval	r2	nsnps
0.001	0.276993544	4.76E-05	868
0.05	0.225849143	7.70E-05	20370
0.1	0.190342257	0.000104537	36492
0.2	0.466033748	9.89E-07	64867
0.3	0.393949365	9.85E-06	89558
0.4	0.473830833	5.86E-07	111582
0.5	0.4296167	4.28E-06	131209

Inositol metabolism - Shin et al., 2014

Chiro-inositol

thresh	pval	r2	nsnps
0.001	0.019721163	0.001655548	868
0.05	0.350257404	5.78E-05	20351
0.1	0.186758993	0.000309299	36460
0.2	0.098767566	0.000648425	64825
0.3	0.091168939	0.000694472	89508
0.4	0.025761242	0.001479844	111521
0.5	0.014034199	0.001882547	131127

myo-inositol

thresh	pval	r2	nsnps
0.001	0.000378464	0.00154137	868
0.05	0.087608676	0.000249864	20368
0.1	0.116720536	0.000193042	36490
0.2	0.382319159	1.22E-05	64866
0.3	0.452661761	1.92E-06	89586
0.4	0.402711422	8.25E-06	111586
0.5	0.430438528	4.18E-06	131210

scyllo-inositol

thresh	pval	r2	nsnps
0.001	0.02383148	0.000640721	868
0.05	0.048613068	0.000449395	20367
0.1	0.038511045	0.000510843	36491
0.2	0.141315768	0.000188639	64866
0.3	0.046482461	0.000461124	89595
0.4	0.029724448	0.000580432	111597
0.5	0.080958653	0.000319646	131214

Sphingomylein (SM) - Rhee et al., 2013 & Draisma et al., 2015 & Shin et al., 2014

SM 14:0

thresh	pval	r2	nsnps
0.001	0.432983553	1.37224E-05	962
0.05	0.351863943	6.96617E-05	23182
0.1	0.166983902	0.000449538	41762
0.2	0.100781943	0.00078534	74775
0.3	0.022839003	0.001921768	103797
0.4	0.008165558	0.00277402	129896
0.5	0.018999245	0.002071594	153317

SM 18:1

thresh	pval	r2	nsnps
0.001	0.001222	0.001226958	939
0.05	0.034565367	0.000441611	22500
0.1	0.153153028	0.00013992	40457
0.2	0.102230353	0.000215276	72185
0.3	0.149801045	0.000143854	99984
0.4	0.140144999	0.000155849	124963
0.5	0.109380768	0.000202209	147254

SM 20:2

thresh	pval	r2	nsnps
0.001	0.11243309	0.000196938	938
0.05	0.014357268	0.00063954	22495
0.1	0.121852539	0.000181697	40444
0.2	0.056411003	0.000336117	72185
0.3	0.122477335	0.000180737	100006
0.4	0.098746275	0.00022204	124988
0.5	0.075188649	0.000276525	147263

SM 22:0

thresh	pval	r2	nsnps
0.001	0.09035357	0.000862729	962
0.05	0.257350653	0.000204458	23182
0.1	0.211183374	0.000310029	41762
0.2	0.278050981	0.000166893	74775
0.3	0.393627839	3.50827E-05	103797
0.4	0.365951038	5.65361E-05	129896
0.5	0.424743802	1.73474E-05	153317

SM 22:1

thresh	pval	r2	nsnps
0.001	0.389907591	3.76456E-05	962
0.05	0.035108613	0.001577721	23182
0.1	0.109239567	0.000729181	41762
0.2	0.127173473	0.000625656	74775
0.3	0.118747924	0.000671931	103797
0.4	0.136760952	0.000577386	129896
0.5	0.12467169	0.000638996	153317

SM 24:0

thresh	pval	r2	nsnps
0.001	0.03347998	0.000448744	939
0.05	0.001680486	0.00114958	22493
0.1	0.000548241	0.001424305	40454
0.2	0.00038748	0.001510196	72171
0.3	0.001051488	0.001264068	100008
0.4	0.000428861	0.00148505	124992
0.5	0.000174927	0.001708131	147285

SM 24:1

thresh	pval	r2	nsnps
0.001	0.057353712	0.000332672	940
0.05	0.024773817	0.000515607	22493
0.1	0.030681954	0.000467948	40446
0.2	0.012264873	0.000675993	72169
0.3	0.034863424	0.000439785	100001
0.4	0.021370494	0.000548875	124966
0.5	0.016336305	0.000609984	147263

SM 26:0

thresh	pval	r2	nsnps
0.001	0.088693822	0.000276925	939
0.05	1.60483E-13	0.008047144	22422
0.1	1.2454E-18	0.011541457	40286
0.2	5.00895E-24	0.015232308	71913
0.3	4.77686E-24	0.015246396	99606
0.4	3.22416E-26	0.016730074	124475
0.5	4.20762E-27	0.017334241	146709

SM 26:1

thresh	pval	r2	nsnps
0.001	0.473919366	5.72718E-07	940
0.05	0.000267835	0.001602783	22495
0.1	0.000292983	0.001580449	40428
0.2	0.000145529	0.001755059	72128
0.3	7.43965E-05	0.001923401	99959
0.4	5.31703E-06	0.002591845	124904
0.5	9.73696E-06	0.002437819	147157

SM 16:0

thresh	pval	r2	nsnps
0.001	0.026532759	0.000507861	868
0.05	0.151148178	0.000144442	20369
0.1	0.303373976	3.60E-05	36491
0.2	0.161667977	0.000132411	64866
0.3	0.169263736	0.000124347	89588
0.4	0.23415721	7.14E-05	111585
0.5	0.337769606	2.38E-05	131216

Cholesterol ester (CE) - Rhee et al., 2013

CE 14:0

thresh	pval	r2	nsnps
0.001	0.223387481	0.000278776	962
0.05	0.039426313	0.001486559	23182
0.1	0.003393776	0.003523808	41762
0.2	0.002616864	0.003748666	74775
0.3	0.001636168	0.004157538	103797
0.4	0.001437732	0.004270648	129896
0.5	0.004339532	0.003312279	153317

CE 16:0

thresh	pval	r2	nsnps
0.001	0.281264835	0.00016152	962
0.05	0.007241882	0.002875531	23182
0.1	0.000721444	0.004877353	41762
0.2	0.001532805	0.004214601	74775
0.3	0.00123457	0.004404204	103797
0.4	0.001174006	0.004448365	129896
0.5	0.001331904	0.00433764	153317

CE 16:1:PC1

thresh	pval	r2	nsnps
0.001	0.365590873	5.68526E-05	962
0.05	0.125219075	0.000636049	23182
0.1	0.044275678	0.001396189	41762
0.2	0.019595453	0.002046349	74775
0.3	0.023384569	0.001902655	103797
0.4	0.026668558	0.00179671	129896
0.5	0.036459084	0.001547971	153317

CE 18:0

thresh	pval	r2	nsnps
0.001	0.180442398	0.000402044	962
0.05	0.083973039	0.000915372	23182
0.1	0.05788709	0.001190802	41762
0.2	0.016594677	0.002182578	74775
0.3	0.010697756	0.002546985	103797
0.4	0.008767713	0.002714026	129896
0.5	0.005819204	0.003061363	153317

CE 18:1

thresh	pval	r2	nsnps
0.001	0.475189755	1.86541E-06	962
0.05	0.150511834	0.000515115	23182
0.1	0.023598717	0.001895281	41762
0.2	0.019183913	0.002063687	74775
0.3	0.021257534	0.001980012	103797
0.4	0.012844076	0.002394474	129896
0.5	0.014957597	0.002268213	153317

CE 18:2

thresh	pval	r2	nsnps
0.001	0.449094793	7.88574E-06	962
0.05	0.136470302	0.000578786	23182
0.1	0.009286933	0.002665611	41762
0.2	0.019148484	0.002065198	74775
0.3	0.021791738	0.001959843	103797
0.4	0.015617823	0.002232555	129896
0.5	0.021692409	0.001963553	153317

CE 18:3

thresh	pval	r2	nsnps
0.001	0.491652318	2.10935E-07	962
0.05	0.017814903	0.002124299	23182
0.1	0.005239697	0.003150869	41762
0.2	0.009555853	0.002641623	74775
0.3	0.006539457	0.00296208	103797
0.4	0.007682178	0.002825576	129896
0.5	0.004000309	0.003382194	153317

CE 20:3

thresh	pval	r2	nsnps
0.001	0.192160647	0.000364497	962
0.05	0.144763184	0.000540231	23182
0.1	0.09827865	0.000803035	41762
0.2	0.250068714	0.000218976	74775
0.3	0.271482422	0.000178246	103797
0.4	0.228292029	0.000266947	129896
0.5	0.183820482	0.000390879	153317

CE 20:4

thresh	pval	r2	nsnps
0.001	0.259835378	0.000199665	962
0.05	0.382199598	4.32676E-05	23182
0.1	0.169837299	0.000439046	41762
0.2	0.382711594	4.2881E-05	74775
0.3	0.198398946	0.000345802	103797
0.4	0.191709565	0.000365882	129896
0.5	0.226386243	0.000271496	153317

CE 20:5

thresh	pval	r2	nsnps
0.001	0.161387498	0.000470828	962
0.05	9.72727E-05	0.006665091	23182
0.1	4.16873E-06	0.009520326	41762
0.2	4.39339E-06	0.009472457	74775
0.3	1.15604E-06	0.010692201	103797
0.4	1.04726E-06	0.010782643	129896
0.5	8.10168E-07	0.01101767	153317

CE 22:6

thresh	pval	r2	nsnps
0.001	0.227766444	0.000268195	962
0.05	0.003396568	0.003523099	23182
0.1	0.000573032	0.00508114	41762
0.2	0.000257544	0.00579252	74775
0.3	9.95219E-05	0.006644537	103797
0.4	2.25241E-05	0.007986184	129896
0.5	2.61413E-05	0.007851237	153317

Cholesterol - Kettunen et al., 2016

Free.C

thresh	pval	r2	nsnps
0.001	0.169768834	6.75813E-05	1410
0.05	0.343323773	1.20573E-05	34880
0.1	0.485175096	1.02359E-07	62715
0.2	0.24075301	3.67075E-05	111433
0.3	0.122455956	0.000100173	154446
0.4	0.095776142	0.000126364	192519
0.5	0.055001812	0.000189222	226752

Serum.C

thresh	pval	r2	nsnps
0.001	0.063589084	0.000108254	1412
0.05	0.490747688	2.50324E-08	35080
0.1	0.332325622	8.74421E-06	63079
0.2	0.311499167	1.12454E-05	112104
0.3	0.272040206	1.71247E-05	155481
0.4	0.332391067	8.73694E-06	193845
0.5	0.299714117	1.28359E-05	228329

Est.C

thresh	pval	r2	nsnps
0.001	0.314385601	1.73171E-05	1410
0.05	0.375939233	7.40601E-06	34881
0.1	0.326632517	1.4952E-05	62717
0.2	0.391845934	5.58318E-06	111438
0.3	0.489958738	4.69473E-08	154454
0.4	0.419520488	3.05681E-06	192530
0.5	0.392278311	5.5375E-06	226767

Other - Shin et al., 2014

4-androsten-3beta,17beta-diol disulfate 1*

thresh	pval	r2	nsnps
0.001	0.245504088	6.45E-05	868
0.05	4.89E-05	0.002061748	20370
0.1	3.20E-06	0.002765019	36492
0.2	7.59E-06	0.002541258	64868
0.3	9.85E-07	0.003071358	89561
0.4	1.04E-06	0.003056949	111587
0.5	3.75E-07	0.003323069	131216

4-androsten-3beta,17beta-diol disulfate 2*

thresh	pval	r2	nsnps
0.001	0.36685849	1.58E-05	868
0.05	0.000794553	0.001359	20370
0.1	0.006252219	0.0008503	36492
0.2	0.01862657	0.000591507	64866
0.3	0.009903031	0.000740065	89582
0.4	0.009303262	0.000754948	111583
0.5	0.005114448	0.000898899	131209

5alpha-androstan-3beta,17beta-diol disulfate

thresh	pval	r2	nsnps
0.001	0.110972093	0.000215113	868
0.05	0.002273345	0.001160483	20371
0.1	0.000165763	0.001856265	36492
0.2	0.000544325	0.001537665	64867
0.3	0.000318468	0.001680848	89584
0.4	0.000618528	0.001503651	111586
0.5	0.000624002	0.001501308	131213

7-alpha-hydroxy-3-oxo-4-cholestenoate (7-Hoca)

thresh	pval	r2	nsnps
0.001	0.066849109	0.000306569	868
0.05	0.129719974	0.000173365	20370
0.1	0.03280746	0.000461986	36492
0.2	0.043659877	0.000398435	64867
0.3	0.111354514	0.000202683	89588
0.4	0.145312013	0.000152235	111587
0.5	0.113534923	0.000198906	131213

androsterone sulfate

cortisol

cortisone

dehydroisoandrosterone sulfate (DHEA-S)

thresh	pval	r2	nsnps
0.001	0.109276235	0.000206282	868
0.05	0.163559371	0.000130854	20366
0.1	0.182956337	0.0001114	36489
0.2	0.27114584	5.06E-05	64865
0.3	0.162500197	0.000132005	89587
0.4	0.159175237	0.000135683	111584
0.5	0.225656899	7.73E-05	131214

thresh	pval	r2	nsnps
0.001	0.462909299	1.18E-06	868
0.05	0.292033574	4.08E-05	20368
0.1	0.076635141	0.000277582	36489
0.2	0.00442669	0.00093234	64863
0.3	0.001994277	0.001127735	89580
0.4	0.001927038	0.001136206	111581
0.5	0.003630831	0.000980608	131210

thresh	pval	r2	nsnps
0.001	0.371632187	1.50E-05	868
0.05	0.306779943	3.57E-05	20369
0.1	0.290973481	4.25E-05	36493
0.2	0.105837428	0.000218577	64861
0.3	0.035562067	0.000456298	89556
0.4	0.026563653	0.000523953	111580
0.5	0.023022628	0.000557571	131203

thresh	pval	r2	nsnps
0.001	0.322082303	2.90E-05	868
0.05	0.027487006	0.000501218	20370
0.1	0.085984934	0.000253941	36492
0.2	0.140058842	0.000158784	64867
0.3	0.031930517	0.000467378	89583
0.4	0.017026086	0.000611314	111584
0.5	0.018279804	0.000594815	131211

epiandrosterone sulfate

thresh	pval	r2	nsnps
0.001	0.24865173	6.29E-05	868
0.05	0.163724439	0.00013089	20369
0.1	0.146966484	0.000150346	36491
0.2	0.160184449	0.000134777	64867
0.3	0.071365243	0.000293196	89586
0.4	0.06428055	0.000315237	111584
0.5	0.091695286	0.000241567	131215

estrone 3-sulfate

thresh	pval	r2	nsnps
0.001	0.412827873	5.29E-05	870
0.05	0.019453077	0.004640194	20305
0.1	0.006570359	0.006684285	36387
0.2	0.004013568	0.007634189	64699
0.3	0.00149815	0.00956111	89325
0.4	0.002546268	0.008519965	111288
0.5	0.002123321	0.008875562	130837

lathosterol

thresh	pval	r2	nsnps
0.001	0.35530052	2.70E-05	869
0.05	0.004604159	0.001327094	20364
0.1	0.168122638	0.000181048	36485
0.2	0.113278752	0.0002863	64841
0.3	0.150161909	0.000210034	89544
0.4	0.197513925	0.000141641	111537
0.5	0.265465445	7.69E-05	131160

5-alpha-pregnan-3beta,20alpha-disulfate

thresh	pval	r2	nsnps
0.001	0.130320785	0.000498632	867
0.05	0.135498169	0.000477496	20368
0.1	0.112958973	0.00057783	36492
0.2	0.108381415	0.000601189	64861
0.3	0.143918252	0.000445196	89555
0.4	0.115164912	0.000566975	111579
0.5	0.131039622	0.000495636	131191

High density lipoprotein (HDL) - Kettunen et al., 2016

HDL.D

thresh	pval	r2	nsnps
0.001	0.298409888	1.45186E-05	1412
0.05	0.014713189	0.000246039	35046
0.1	0.003994281	0.000365006	63012
0.2	0.000532092	0.000555671	111974
0.3	0.004259802	0.000359032	155283
0.4	0.002641992	0.000403571	193573
0.5	0.003278729	0.000383383	228004

HDL.C

thresh	pval	r2	nsnps
0.001	0.052392908	0.000122059	1412
0.05	0.000189685	0.000585825	35085
0.1	7.51004E-05	0.00066643	63090
0.2	1.02432E-05	0.000841378	112122
0.3	0.000138351	0.000613213	155506
0.4	7.81571E-05	0.000662948	193869
0.5	0.000184277	0.000588332	228354

S.HDL.L

thresh	pval	r2	nsnps
0.001	0.318132621	1.16048E-05	1412
0.05	0.024775499	0.000200083	35047
0.1	0.063422688	0.000120923	63007
0.2	0.069909987	0.000113102	111979
0.3	0.043347405	0.000152258	155287
0.4	0.020029325	0.000218697	193582
0.5	0.027562775	0.000190833	228014

S.HDL.P

thresh	pval	r2	nsnps
0.001	0.176453331	4.47755E-05	1412
0.05	0.025164852	0.000198727	35052
0.1	0.045877653	0.000147517	63023
0.2	0.049323299	0.000141498	112000
0.3	0.036990876	0.000165627	155317
0.4	0.017909183	0.00022857	193619
0.5	0.025229906	0.000198502	228059

S.HDL.TG

thresh	pval	r2	nsnps
0.001	0.193427921	3.47337E-05	1412
0.05	0.438257897	1.12001E-06	35088
0.1	0.152460344	4.88229E-05	63094
0.2	0.21858076	2.80041E-05	112128
0.3	0.357837538	6.15428E-06	155513
0.4	0.398010407	3.09965E-06	193882
0.5	0.482106884	9.33759E-08	228370

M.HDL.C

thresh	pval	r2	nsnps
0.001	0.038628911	0.000144793	1412
0.05	0.000477965	0.000505997	35087
0.1	0.000167144	0.000596713	63095
0.2	3.04689E-05	0.000745305	112124
0.3	0.00028475	0.000550601	155511
0.4	0.000197768	0.000582128	193876
0.5	0.000889972	0.0004528	228362

M.HDL.CE

thresh	pval	r2	nsnps
0.001	0.033443847	0.000174207	1411
0.05	0.005661026	0.000332745	35049
0.1	0.003961355	0.000365775	63015
0.2	0.001472613	0.000458624	111988
0.3	0.007216088	0.000310464	155299
0.4	0.004734263	0.000349249	193595
0.5	0.012641939	0.000259622	228030

M.HDL.FC

thresh	pval	r2	nsnps
0.001	0.137649813	5.52027E-05	1412
0.05	0.000985264	0.000444114	35088
0.1	0.000729956	0.000469695	63094
0.2	0.000163865	0.000598404	112127
0.3	0.001278994	0.000421946	155509
0.4	0.000804525	0.000461388	193877
0.5	0.002639636	0.000360861	228364

M.HDL.L

thresh	pval	r2	nsnps
0.001	0.080501627	0.000101939	1412
0.05	0.001748902	0.000442371	35052
0.1	0.003889429	0.000367477	63023
0.2	0.001063009	0.000489551	112000
0.3	0.00504242	0.000343419	155317
0.4	0.004319084	0.00035775	193619
0.5	0.009735483	0.000283191	228059

M.HDL.P

thresh	pval	r2	nsnps
0.001	0.098286218	8.65245E-05	1412
0.05	0.001509341	0.000456293	35052
0.1	0.004685503	0.000350207	63023
0.2	0.000965533	0.000498705	112000
0.3	0.004490302	0.000354147	155317
0.4	0.003842586	0.000368604	193619
0.5	0.008059877	0.000300363	228059

M.HDL.PL

thresh	pval	r2	nsnps
0.001	0.141183082	5.36059E-05	1412
0.05	0.000374326	0.000527016	35088
0.1	0.000488665	0.000504095	63094
0.2	2.28124E-05	0.000770727	112128
0.3	0.000195838	0.000582978	155513
0.4	0.000156308	0.000602529	193882
0.5	0.000526443	0.000497704	228370

L.HDL.C

thresh	pval	r2	nsnps
0.001	0.088425222	8.46005E-05	1412
0.05	0.001848176	0.000390835	35085
0.1	0.000179304	0.000590622	63090
0.2	4.34811E-05	0.000714123	112118
0.3	0.000780141	0.000464037	155496
0.4	0.000591468	0.000487719	193861
0.5	0.001181592	0.000428687	228343

L.HDL.CE

thresh	pval	r2	nsnps
0.001	0.09018167	9.31118E-05	1412
0.05	0.006564726	0.00031913	35052
0.1	0.001485269	0.000457814	63023
0.2	0.000860471	0.000509685	112000
0.3	0.009330039	0.000287049	155317
0.4	0.006293689	0.000323001	193619
0.5	0.010181875	0.000279131	228059

L.HDL.FC

thresh	pval	r2	nsnps
0.001	0.219531576	2.77713E-05	1412
0.05	0.0042417	0.000321321	35087
0.1	0.000349699	0.000532853	63092
0.2	5.16574E-05	0.000699006	112127
0.3	0.000588062	0.000488191	155512
0.4	0.000273303	0.000554118	193881
0.5	0.000552424	0.000493549	228367

L.HDL.L

thresh	pval	r2	nsnps
0.001	0.14437416	5.83934E-05	1412
0.05	0.006785176	0.000316102	35052
0.1	0.001917531	0.000433689	63023
0.2	0.000309927	0.00060768	112000
0.3	0.003698428	0.00037216	155317
0.4	0.00204165	0.000427781	193619
0.5	0.003604272	0.00037456	228059

L.HDL.P

thresh	pval	r2	nsnps
0.001	0.14730247	5.6993E-05	1411
0.05	0.007184276	0.000310868	35053
0.1	0.00208924	0.000425613	63023
0.2	0.000233827	0.000634905	111997
0.3	0.002942748	0.00039348	155311
0.4	0.001732028	0.000443286	193607
0.5	0.003132041	0.000387655	228046

L.HDL.PL

thresh	pval	r2	nsnps
0.001	0.162037715	5.04542E-05	1412
0.05	0.004879768	0.000346449	35052
0.1	0.00128502	0.000471536	63023
0.2	0.00011154	0.000706747	112000
0.3	0.001393985	0.00046382	155317
0.4	0.000653346	0.000535997	193619
0.5	0.001377793	0.000464927	228059

XL.HDL.C

thresh	pval	r2	nsnps
0.001	0.145529422	5.17497E-05	1412
0.05	0.021905045	0.000188648	35088
0.1	0.006902434	0.000281433	63093
0.2	0.012117107	0.00023571	112126
0.3	0.047605396	0.000129239	155509
0.4	0.051683737	0.000123149	193875
0.5	0.032370046	0.000158371	228362

XL.HDL.CE

thresh	pval	r2	nsnps
0.001	0.17474898	4.54138E-05	1412
0.05	0.033352006	0.000174442	35047
0.1	0.015169934	0.000243312	63016
0.2	0.038411614	0.000162435	111980
0.3	0.136074726	6.25664E-05	155290
0.4	0.133804035	6.37642E-05	193587
0.5	0.099081673	8.59123E-05	228017

XL.HDL.FC

thresh	pval	r2	nsnps
0.001	0.140293137	5.4044E-05	1412
0.05	0.006485232	0.000286522	35088
0.1	0.000937357	0.000448712	63094
0.2	0.000687617	0.000475178	112128
0.3	0.003766027	0.00033146	155513
0.4	0.004279833	0.000320832	193882
0.5	0.002966698	0.000351364	228369

XL.HDL.L

thresh	pval	r2	nsnps
0.001	0.333182326	9.64474E-06	1411
0.05	0.041256953	0.000156407	35050
0.1	0.007748542	0.000303957	63013
0.2	0.013740024	0.000252155	111976
0.3	0.066164776	0.000117513	155278

XL.HDL.P

thresh	pval	r2	nsnps
0.001	0.393477951	3.78993E-06	1412
0.05	0.021074231	0.000214227	35052
0.1	0.001488101	0.000457634	63023
0.2	0.000578825	0.000547598	112000
0.3	0.00260638	0.000404842	155317

XL.HDL.PL

thresh	pval	r2	nsnps
0.001	0.305923554	1.33603E-05	1412
0.05	0.025967276	0.000196	35052
0.1	0.003535757	0.000376348	63023
0.2	0.002585328	0.000405603	112000
0.3	0.01636236	0.000236579	155317

XL.HDL.TG

thresh	pval	r2	nsnps
0.001	0.441038155	1.02173E-06	1412
0.05	0.254061132	2.03347E-05	35088
0.1	0.335409282	8.38806E-06	63094
0.2	0.338064512	8.10343E-06	112128
0.3	0.321184515	1.00137E-05	155513

0.4	0.057900536	0.000128317	193569
0.5	0.04355814	0.000151852	228002

0.4	0.001356104	0.000466431	193619
0.5	0.000866977	0.000508966	228059

0.4	0.01174052	0.000266273	193619
0.5	0.009785217	0.000282729	228059

0.4	0.362904643	5.71109E-06	193882
0.5	0.391887039	3.49635E-06	228369

Intermediate density lipoprotein (IDL) - Kettunen et al., 2016

IDL.C

thresh	pval	r2	nsnps
0.001	0.031795622	0.000178532	1412
0.05	0.206681483	3.47167E-05	35052
0.1	0.1148595	7.4848E-05	63023
0.2	0.180127061	4.34281E-05	112000
0.3	0.178633673	4.39712E-05	155317
0.4	0.170108487	4.71951E-05	193619
0.5	0.156883629	5.26501E-05	228059

IDL.FC

thresh	pval	r2	nsnps
0.001	0.009183582	0.0002579	1412
0.05	0.281138764	1.55746E-05	35084
0.1	0.148815151	5.03156E-05	63084
0.2	0.273146566	1.68838E-05	112114
0.3	0.270232464	1.73791E-05	155496
0.4	0.305639389	1.19818E-05	193858
0.5	0.296927888	1.31899E-05	228342

IDL.L

thresh	pval	r2	nsnps
0.001	0.043489118	0.000151985	1412
0.05	0.293784183	1.52626E-05	35052
0.1	0.156323173	5.28945E-05	63023
0.2	0.277001487	1.81701E-05	112000
0.3	0.268763301	1.97239E-05	155317
0.4	0.266781218	2.0111E-05	193619
0.5	0.258346173	2.18174E-05	228059

IDL.P

thresh	pval	r2	nsnps
0.001	0.050467457	0.000139601	1412
0.05	0.332860874	9.68435E-06	35052
0.1	0.17503497	4.53061E-05	63023
0.2	0.316870045	1.17793E-05	112000
0.3	0.300928458	1.41235E-05	155317
0.4	0.309939856	1.27658E-05	193619
0.5	0.308964824	1.29086E-05	228059

IDL.PL

thresh	pval	r2	nsnps
0.001	0.01996059	0.00019578	1412
0.05	0.286793099	1.46907E-05	35088
0.1	0.129134898	5.92778E-05	63094
0.2	0.306418815	1.18773E-05	112128
0.3	0.328639228	9.13055E-06	155513
0.4	0.378018417	4.47737E-06	193882
0.5	0.377093088	4.54783E-06	228370

IDL.TG

thresh	pval	r2	nsnps
0.001	0.218286925	3.14046E-05	1412
0.05	0.435900101	1.35116E-06	35052
0.1	0.231293183	2.79988E-05	63023
0.2	0.406585921	2.89808E-06	112000
0.3	0.376003765	5.1809E-06	155317
0.4	0.382568616	4.63064E-06	193619
0.5	0.395485307	3.64508E-06	228059

Low density lipoprotein (LDL) - Kettunen et al., 2016

LDL.D

thresh	pval	r2	nsnps
0.001	0.151008244	5.52711E-05	1412
0.05	0.248637133	2.39061E-05	35052
0.1	0.449682474	8.29816E-07	63023
0.2	0.148529958	5.64165E-05	112000
0.3	0.152527976	5.45806E-05	155317
0.4	0.164815907	4.93084E-05	193619
0.5	0.187025267	4.09977E-05	228059

LDL.C

thresh	pval	r2	nsnps
0.001	0.089353497	8.38753E-05	1412
0.05	0.213492229	2.92686E-05	35088
0.1	0.128472216	5.96089E-05	63094
0.2	0.066715695	0.000104458	112128
0.3	0.081522882	9.02482E-05	155513
0.4	0.092283805	8.16543E-05	193882
0.5	0.105573668	7.25212E-05	228370

S.LDL.C

thresh	pval	r2	nsnps
0.001	0.063045159	0.000108548	1412
0.05	0.391737436	3.50303E-06	35088
0.1	0.146996728	5.10862E-05	63094
0.2	0.060863106	0.000111098	112128
0.3	0.078338643	9.30559E-05	155513
0.4	0.079212511	9.22761E-05	193882
0.5	0.093400023	8.08409E-05	228370

S.LDL.L

thresh	pval	r2	nsnps
0.001	0.077457521	0.000104969	1412
0.05	0.312140116	1.24473E-05	35052
0.1	0.178360482	4.40713E-05	63021
0.2	0.105558689	8.11334E-05	111998
0.3	0.109739476	7.82294E-05	155316
0.4	0.122196081	7.03072E-05	193618
0.5	0.141170685	5.99673E-05	228057

S.LDL.P

thresh	pval	r2	nsnps
0.001	0.151559382	5.50196E-05	1412
0.05	0.477164449	1.70188E-07	35052
0.1	0.277069051	1.81577E-05	63023
0.2	0.191892807	3.93569E-05	112000
0.3	0.185651221	4.14717E-05	155317
0.4	0.195760448	3.80947E-05	193619
0.5	0.219617632	3.10419E-05	228059

M.LDL.C

thresh	pval	r2	nsnps
0.001	0.140808524	5.37705E-05	1412
0.05	0.382927718	4.11359E-06	35086
0.1	0.278056771	1.6071E-05	63088
0.2	0.151524937	4.91998E-05	112126
0.3	0.172483138	4.13688E-05	155509
0.4	0.197056384	3.36844E-05	193875
0.5	0.21354319	2.92557E-05	228365

M.LDL.CE

thresh	pval	r2	nsnps
0.001	0.061309922	0.000123665	1412
0.05	0.289923557	1.5902E-05	35052
0.1	0.154782829	5.35724E-05	63023
0.2	0.079273923	0.000103145	112000
0.3	0.090177037	9.31158E-05	155317
0.4	0.101683158	8.39498E-05	193619
0.5	0.107626752	7.96801E-05	228059

M.LDL.L

thresh	pval	r2	nsnps
0.001	0.058360999	0.000127671	1412
0.05	0.251791573	2.32124E-05	35051
0.1	0.154664613	5.36248E-05	63022
0.2	0.096085658	8.82487E-05	111996
0.3	0.108801269	7.88695E-05	155313
0.4	0.123960369	6.92646E-05	193614
0.5	0.135671688	6.27772E-05	228054

M.LDL.P

thresh	pval	r2	nsnps
0.001	0.07046738	0.000112468	1412
0.05	0.280773431	1.74872E-05	35051
0.1	0.167296109	4.8307E-05	63021
0.2	0.113740347	7.55715E-05	111997
0.3	0.124712592	6.88255E-05	155312
0.4	0.135254803	6.2996E-05	193613
0.5	0.153453482	5.41645E-05	228052

M.LDL.PL

thresh	pval	r2	nsnps
0.001	0.224103154	2.66804E-05	1412
0.05	0.428980621	1.48577E-06	35088
0.1	0.297774555	1.30698E-05	63094
0.2	0.233000406	2.46515E-05	112128
0.3	0.251149742	2.08772E-05	155513
0.4	0.261360609	1.89497E-05	193882
0.5	0.280936023	1.56077E-05	228370

L.LDL.C

thresh	pval	r2	nsnps
0.001	0.04345399	0.000135975	1412
0.05	0.179481819	3.90451E-05	35088
0.1	0.118442349	6.49124E-05	63094
0.2	0.107070124	7.16016E-05	112128
0.3	0.123546386	6.21578E-05	155513
0.4	0.139620246	5.43221E-05	193882
0.5	0.133795619	5.70257E-05	228370

L.LDL.CE

thresh	pval	r2	nsnps
0.001	0.058921546	0.000126893	1412
0.05	0.258097159	2.18692E-05	35052
0.1	0.148862463	5.62614E-05	63023
0.2	0.101155081	8.43434E-05	112000
0.3	0.113000484	7.60545E-05	155317
0.4	0.116807945	7.36085E-05	193619
0.5	0.115256435	7.45935E-05	228059

L.LDL.FC

L.LDL.L

L.LDL.P

L.LDL.PL

thresh	pval	r2	nsnps
0.001	0.024335161	0.000180299	1412
0.05	0.209440656	3.03151E-05	35088
0.1	0.169649166	4.23596E-05	63094
0.2	0.246655901	2.17717E-05	112128
0.3	0.283146468	1.52597E-05	155513
0.4	0.304665511	1.21155E-05	193882
0.5	0.301277174	1.25799E-05	228370

thresh	pval	r2	nsnps
0.001	0.049691055	0.000140883	1412
0.05	0.251472406	2.32819E-05	35052
0.1	0.150601435	5.54574E-05	63023
0.2	0.146716254	5.72705E-05	112000
0.3	0.154487909	5.37032E-05	155317
0.4	0.160935172	5.09161E-05	193619
0.5	0.165075358	4.92027E-05	228059

thresh	pval	r2	nsnps
0.001	0.057144818	0.000129389	1412
0.05	0.275602601	1.84278E-05	35052
0.1	0.171003212	4.68466E-05	63023
0.2	0.18425572	4.19582E-05	112000
0.3	0.19018477	3.99259E-05	155317
0.4	0.198078017	3.73553E-05	193619
0.5	0.20597926	3.4926E-05	228059

thresh	pval	r2	nsnps
0.001	0.034434164	0.000153575	1412
0.05	0.222880416	2.69791E-05	35088
0.1	0.150561248	4.96144E-05	63094
0.2	0.248768227	2.13542E-05	112128
0.3	0.291104482	1.40452E-05	155513
0.4	0.307045544	1.17986E-05	193882
0.5	0.302707484	1.23853E-05	228370

Very low density lipoprotein (VLDL) - Kettunen et al., 2016

VLDL.D

thresh	pval	r2	nsnps
0.001	0.216325731	3.19455E-05	1412
0.05	0.194726661	3.84286E-05	35052
0.1	0.372023578	5.53111E-06	63023
0.2	0.102828539	8.31044E-05	112000
0.3	0.055601427	0.000131629	155317
0.4	0.044629136	0.00014982	193619
0.5	0.068449229	0.00011479	228059

XS.VLDL.L

thresh	pval	r2	nsnps
0.001	0.065483009	0.000118346	1412
0.05	0.322403954	1.10262E-05	35052
0.1	0.088447328	9.46109E-05	63023
0.2	0.199365107	3.69501E-05	112000
0.3	0.199754985	3.68281E-05	155317
0.4	0.214915194	3.23391E-05	193619
0.5	0.201714718	3.622E-05	228059

XS.VLDL.P

thresh	pval	r2	nsnps
0.001	0.071968113	0.00011079	1412
0.05	0.410883114	2.63313E-06	35045
0.1	0.108686018	7.89486E-05	63012
0.2	0.250732671	2.34436E-05	111973
0.3	0.242369017	2.53297E-05	155277
0.4	0.276713258	1.8223E-05	193566
0.5	0.271106221	1.92731E-05	227993

XS.VLDL.PL

thresh	pval	r2	nsnps
0.001	0.022551996	0.000208288	1412
0.05	0.310529567	1.26799E-05	35049
0.1	0.134909717	6.31778E-05	63019
0.2	0.309548237	1.2823E-05	111994
0.3	0.300348643	1.42139E-05	155304
0.4	0.33186546	9.80762E-06	193597
0.5	0.319314964	1.14428E-05	228035

XS.VLDL.TG

thresh	pval	r2	nsnps
0.001	0.243584926	2.50488E-05	1412
0.05	0.433291116	1.46445E-06	35052
0.1	0.146967668	5.71513E-05	63023
0.2	0.139029047	6.1045E-05	112000
0.3	0.145758601	5.77268E-05	155317
0.4	0.153375796	5.41993E-05	193619
0.5	0.169356547	4.749E-05	228059

S.VLDL.C

thresh	pval	r2	nsnps
0.001	0.060689447	0.0001113	1412
0.05	0.097488804	7.79113E-05	35080
0.1	0.042083761	0.000138346	63079
0.2	0.016203942	0.000212289	112099
0.3	0.026115709	0.000174793	155476
0.4	0.040794406	0.000140685	193841
0.5	0.032157531	0.000158751	228319

S.VLDL.FC

thresh	pval	r2	nsnps
0.001	0.168790949	4.26538E-05	1412
0.05	0.196485168	3.38475E-05	35083
0.1	0.045686242	0.000132188	63085
0.2	0.010850021	0.000244401	112110
0.3	0.013643964	0.000225982	155485
0.4	0.017586773	0.000205774	193846
0.5	0.014491883	0.000221163	228324

S.VLDL.L

thresh	pval	r2	nsnps
0.001	0.200064385	3.67315E-05	1412
0.05	0.270038124	1.94778E-05	35052
0.1	0.093301654	9.04973E-05	63023
0.2	0.022434922	0.000208743	111994
0.3	0.025178828	0.000198678	155310
0.4	0.026342204	0.000194756	193609
0.5	0.027695707	0.000190417	228050

S.VLDL.P

thresh	pval	r2	nsnps
0.001	0.254046792	2.27254E-05	1412
0.05	0.300137815	1.42468E-05	35052
0.1	0.091351561	9.21193E-05	63023
0.2	0.018690652	0.000224795	112000
0.3	0.020859666	0.000215125	155317
0.4	0.02087652	0.000215054	193619
0.5	0.023334481	0.000205307	228059

S.VLDL.PL

thresh	pval	r2	nsnps
0.001	0.120142382	6.39821E-05	1412
0.05	0.257672684	1.96369E-05	35088
0.1	0.096460633	7.86553E-05	63096
0.2	0.036183916	0.000149795	112129
0.3	0.048362752	0.000128001	155515
0.4	0.061596521	0.000110256	193880
0.5	0.055188494	0.000118262	228364

S.VLDL.TG

thresh	pval	r2	nsnps
0.001	0.428385397	1.51105E-06	1412
0.05	0.321528015	9.96226E-06	35085
0.1	0.047178608	0.0001298	63085
0.2	0.005886959	0.00029426	112120
0.3	0.008318206	0.00026596	155502
0.4	0.00889838	0.000260475	193861
0.5	0.012138117	0.000235374	228342

M.VLDL.C

thresh	pval	r2	nsnps
0.001	0.334903572	8.43706E-06	1412
0.05	0.418315088	1.97303E-06	35088
0.1	0.114972062	6.6872E-05	63094
0.2	0.03911506	0.000143894	112128
0.3	0.034791978	0.00015278	155513
0.4	0.034894369	0.000152556	193882
0.5	0.037058004	0.000147983	228370

M.VLDL.CE

thresh	pval	r2	nsnps
0.001	0.259310189	2.16174E-05	1412
0.05	0.326973286	1.0427E-05	35052
0.1	0.104268914	8.20569E-05	63023
0.2	0.029202787	0.000185843	112000
0.3	0.021828145	0.000211144	155317
0.4	0.016949991	0.000233447	193619
0.5	0.018887783	0.000223868	228059

M.VLDL.FC

thresh	pval	r2	nsnps
0.001	0.410923079	2.3871E-06	1412
0.05	0.493165264	1.38201E-08	35067
0.1	0.153449731	4.915E-05	63038
0.2	0.040826341	0.000142725	112021
0.3	0.037243164	0.000149767	155350
0.4	0.051033019	0.000125838	193675
0.5	0.054609659	0.000120778	228136

M.VLDL.L

thresh	pval	r2	nsnps
0.001	0.285492953	1.66571E-05	1412
0.05	0.360043012	6.66268E-06	35052
0.1	0.272399971	1.90273E-05	63023
0.2	0.09618586	8.81692E-05	112000
0.3	0.091569855	9.19358E-05	155317
0.4	0.097271036	8.73143E-05	193619
0.5	0.121801476	7.0543E-05	228059

M.VLDL.P

thresh	pval	r2	nsnps
0.001	0.324985596	1.06852E-05	1412
0.05	0.340957231	8.71572E-06	35052
0.1	0.082368036	0.000100146	63023
0.2	0.005068347	0.000342945	112000
0.3	0.002553552	0.000406762	155319
0.4	0.00218951	0.000421203	193621
0.5	0.00491533	0.000345778	228060

M.VLDL.TG

thresh	pval	r2	nsnps
0.001	0.246250671	2.21765E-05	1412
0.05	0.433840156	1.30679E-06	35067
0.1	0.166766215	4.4024E-05	63038
0.2	0.041359273	0.000141728	112021
0.3	0.045188754	0.000134996	155350
0.4	0.079476614	9.34073E-05	193675

L.VLDL.C

thresh	pval	r2	nsnps
0.001	0.161358336	4.60505E-05	1412
0.05	0.291665915	1.41689E-05	35057
0.1	0.326928083	9.4689E-06	63013
0.2	0.191003497	3.59887E-05	111977
0.3	0.145882684	5.23395E-05	155298
0.4	0.180179065	3.93986E-05	193606

L.VLDL.CE

thresh	pval	r2	nsnps
0.001	0.22286003	3.067E-05	1411
0.05	0.429459057	1.66643E-06	35018
0.1	0.220107996	3.14193E-05	62951
0.2	0.042390299	0.000156675	111853
0.3	0.028090692	0.000192317	155099
0.4	0.028974874	0.000189598	193352

L.VLDL.FC

thresh	pval	r2	nsnps
0.001	0.053541312	0.000122262	1412
0.05	0.202120014	3.27557E-05	35067
0.1	0.414633516	2.18953E-06	63038
0.2	0.211205809	3.03035E-05	112021
0.3	0.150589636	5.03314E-05	155350
0.4	0.195353719	3.46891E-05	193675

0.5	0.102883704	7.53683E-05	228136

0.5	0.169204843	4.31564E-05	228050

0.5	0.035375617	0.000172216	227754

0.5	0.171884441	4.2203E-05	228136

L.VLDL.L

thresh	pval	r2	nsnps
0.001	0.05785503	0.0001305	1411
0.05	0.147562559	5.78091E-05	35023
0.1	0.458386095	5.75968E-07	62957
0.2	0.288275597	1.64473E-05	111867
0.3	0.199913758	3.73856E-05	155122
0.4	0.264138286	2.09758E-05	193375
0.5	0.296942023	1.49956E-05	227784

L.VLDL.P

thresh	pval	r2	nsnps
0.001	0.078163651	0.000105975	1411
0.05	0.316922046	1.19664E-05	35023
0.1	0.112139367	7.78863E-05	62957
0.2	0.011671595	0.000271207	111867
0.3	0.002455303	0.000417216	155122
0.4	0.002525618	0.000414524	193375
0.5	0.003390411	0.000386535	227784

L.VLDL.PL

thresh	pval	r2	nsnps
0.001	0.155441629	4.83495E-05	1412
0.05	0.164388029	4.49028E-05	35052
0.1	0.465470655	3.53597E-07	63011
0.2	0.32247093	9.99745E-06	111973
0.3	0.298524912	1.31582E-05	155285
0.4	0.422634081	1.79327E-06	193582
0.5	0.4262828	1.62619E-06	228036

L.VLDL.TG

thresh	pval	r2	nsnps
0.001	0.043211946	0.000138403	1412
0.05	0.216889358	2.88467E-05	35065
0.1	0.330297655	9.07766E-06	63036
0.2	0.157659174	4.74713E-05	112017
0.3	0.100130183	7.72358E-05	155342
0.4	0.166775816	4.40246E-05	193665
0.5	0.187827424	3.69537E-05	228125

XL.VLDL.L

thresh	pval	r2	nsnps
0.001	0.024509004	0.000201025	1412
0.05	0.199335435	3.69594E-05	35046
0.1	0.439455945	1.20428E-06	63017
0.2	0.228892432	2.86046E-05	111985
0.3	0.130236511	6.56981E-05	155295
0.4	0.129194508	6.62754E-05	193591
0.5	0.114633634	7.49934E-05	228025

XL.VLDL.P

thresh	pval	r2	nsnps
0.001	0.158537748	5.27925E-05	1385
0.05	0.337508748	9.27152E-06	34281
0.1	0.32934493	1.0291E-05	61613
0.2	0.060536231	0.000126753	109522
0.3	0.031641521	0.000181902	151928
0.4	0.051462088	0.000140266	189392
0.5	0.046584339	0.000148657	223084

XL.VLDL.PL

thresh	pval	r2	nsnps
0.001	0.071338206	0.000101179	1412
0.05	0.292930517	1.39781E-05	35068
0.1	0.386709786	3.90305E-06	63039
0.2	0.20974336	3.0689E-05	112018
0.3	0.129817927	5.98323E-05	155350
0.4	0.157530996	4.75261E-05	193672
0.5	0.150196337	5.04979E-05	228129

XL.VLDL.TG

thresh	pval	r2	nsnps
0.001	0.059561949	0.00011271	1412
0.05	0.217471667	2.82893E-05	35071
0.1	0.413123402	2.23631E-06	63066
0.2	0.223005345	2.6952E-05	112069
0.3	0.107188619	7.15408E-05	155422
0.4	0.153082014	4.8595E-05	193773
0.5	0.129270289	5.92406E-05	228241

XXL.VLDL.L

thresh	pval	r2	nsnps
0.001	0.052788565	0.000138134	1411
0.05	0.164162289	5.0394E-05	35023
0.1	0.40638667	2.95874E-06	62957
0.2	0.43696425	1.32786E-06	111867
0.3	0.362672906	6.5099E-06	155122
0.4	0.32307879	1.1117E-05	193375
0.5	0.329370664	1.02877E-05	227784

XXL.VLDL.P

thresh	pval	r2	nsnps
0.001	0.090761247	9.41472E-05	1397
0.05	0.035459284	0.000172012	34810
0.1	0.094092035	9.13343E-05	62592
0.2	0.156849974	5.35341E-05	111254
0.3	0.253293544	2.3265E-05	154273
0.4	0.263150949	2.11773E-05	192326
0.5	0.2136261	3.32421E-05	226559

XXL.VLDL.PL

thresh	pval	r2	nsnps
0.001	0.050051433	0.00012551	1412
0.05	0.292301632	1.38729E-05	35088
0.1	0.397818231	3.11392E-06	63094
0.2	0.318352303	1.03554E-05	112128
0.3	0.257693942	1.96411E-05	155513
0.4	0.225427969	2.63899E-05	193882
0.5	0.211271189	2.98591E-05	228369

XXL.VLDL.TG

thresh	pval	r2	nsnps
0.001	0.226468969	2.61505E-05	1412
0.05	0.361097802	5.86807E-06	35081
0.1	0.092594033	8.14957E-05	63080
0.2	0.052036847	0.000122647	112102
0.3	0.034741501	0.000152968	155474
0.4	0.030601656	0.000162683	193830
0.5	0.037445761	0.000147269	228304

Apolipoproteins

ApoA1

thresh	pval	r2	nsnps
0.001	0.072363307	0.000102811	1411
0.05	0.000127215	0.000646535	35037
0.1	0.002583171	0.000377957	62992
0.2	0.000838138	0.000477192	111963
0.3	0.007394071	0.00028717	155257
0.4	0.008609489	0.000274237	193517
0.5	0.012372649	0.00024368	227941

ApoB

thresh	pval	r2	nsnps
0.001	0.073889422	0.000101253	1412
0.05	0.110136159	7.26216E-05	35069
0.1	0.021687326	0.000197213	63057
0.2	0.0054798	0.000312752	112075
0.3	0.005543492	0.00031176	155425
0.4	0.003496152	0.000351555	193756
0.5	0.002953447	0.00036622	228225

Bile acid metabolism - Shin et al., 2014

cholate

thresh	pval	r2	nsnps
0.001	0.270019049	6.71E-05	867
0.05	0.189436222	0.000138293	20361
0.1	0.398608774	1.18E-05	36487
0.2	0.250018542	8.12E-05	64857
0.3	0.276473427	6.29E-05	89576
0.4	0.198245953	0.000128397	111574
0.5	0.087691659	0.000327918	131188

deoxycholate

thresh	pval	r2	nsnps
0.001	0.139565761	0.000237368	869
0.05	0.46904007	1.22E-06	20358
0.1	0.419289853	8.41E-06	36480
0.2	0.455127005	2.58E-06	64862
0.3	0.16151186	0.000197926	89573
0.4	0.097335776	0.000340822	111569
0.5	0.078177683	0.000407117	131178

glycochenodeoxycholate

thresh	pval	r2	nsnps
0.001	0.027474198	0.000550716	868
0.05	0.485057165	2.10E-07	20370
0.1	0.422938589	5.65E-06	36491
0.2	0.359828124	1.93E-05	64872
0.3	0.455711528	1.85E-06	89568
0.4	0.460167223	1.50E-06	111591
0.5	0.278280216	5.17E-05	131217

glycocholate

thresh	pval	r2	nsnps
0.001	0.006413512	0.001100534	869
0.05	0.328844107	3.49E-05	20363
0.1	0.38837596	1.43E-05	36485
0.2	0.45650703	2.12E-06	64863
0.3	0.43341901	5.00E-06	89569
0.4	0.390294109	1.38E-05	111567
0.5	0.468390575	1.12E-06	131186

glycodeoxycholate

thresh	pval	r2	nsnps
0.001	0.194049854	0.000511117	871
0.05	0.031281136	0.002377487	20367
0.1	0.006376257	0.004248412	36482
0.2	0.006335734	0.004256125	64852
0.3	0.006672574	0.004193496	89550
0.4	0.003381652	0.005021514	111545
0.5	0.001922054	0.005718674	131157

hyodeoxycholate

thresh	pval	r2	nsnps
0.001	0.276971606	6.17E-05	868
0.05	0.063056438	0.000412344	20371
0.1	0.008635283	0.000998737	36499
0.2	0.009553754	0.000967564	64871
0.3	0.005165641	0.001158655	89583
0.4	0.001237735	0.001613123	111586
0.5	0.001254704	0.001608738	131199

taurochenodeoxycholate

thresh	pval	r2	nsnps
0.001	0.455643959	2.31E-06	869
0.05	0.179106713	0.000157009	20368
0.1	0.255296032	8.05E-05	36492
0.2	0.12004032	0.000256686	64873
0.3	0.058817442	0.000455352	89565
0.4	0.073154621	0.000392467	111592
0.5	0.165619812	0.000175588	131221

taurocholate

thresh	pval	r2	nsnps
0.001	0.361598889	3.32E-05	866
0.05	0.312641107	6.30E-05	20352
0.1	0.177105292	0.000226862	36472
0.2	0.035163323	0.000865441	64860
0.3	0.028866304	0.00095153	89558
0.4	0.016857575	0.001191225	111593
0.5	0.036093093	0.000854141	131221

taurodeoxycholate

thresh	pval	r2	nsnps
0.001	0.32377736	0.000135439	867
0.05	0.306720845	0.000165374	20365
0.1	0.122871495	0.000872817	36495
0.2	0.189110212	0.0005031	64863
0.3	0.22711196	0.000362922	89558
0.4	0.122435664	0.000876045	111585
0.5	0.11997301	0.000894552	131203

taurolithocholate 3-sulfate

thresh	pval	r2	nsnps
0.001	0.018994862	0.000661044	868
0.05	0.079284086	0.000305256	20365
0.1	0.062831491	0.000360138	36487
0.2	0.005315796	0.001001783	64862
0.3	0.008924248	0.000861395	89555
0.4	0.009176354	0.000853907	111591
0.5	0.00556821	0.000989131	131213

ursodeoxycholate

thresh	pval	r2	nsnps
0.001	0.283629511	6.34E-05	868
0.05	0.348586042	2.93E-05	20361
0.1	0.313946687	4.55E-05	36491
0.2	0.302874707	5.16E-05	64860
0.3	0.37253987	2.05E-05	89539
0.4	0.425405373	6.85E-06	111554
0.5	0.350200218	2.87E-05	131167

Other - Kettunen et al., 2016 & Shin et al., 2014

Alb (Albumin)

thresh	pval	r2	nsnps
0.001	0.143454972	5.98118E-05	1411
0.05	0.359996359	6.77739E-06	35021
0.1	0.27392768	1.9049E-05	62951
0.2	0.269554949	1.98939E-05	111861
0.3	0.310570417	1.28832E-05	155111
0.4	0.210921359	3.40277E-05	193360
0.5	0.141782476	6.06482E-05	227766

Glol (glycerol)

thresh	pval	r2	nsnps
0.001	0.10447976	7.80113E-05	1412
0.05	0.347070659	7.64211E-06	34956
0.1	0.477150689	1.62292E-07	62873
0.2	0.291038852	1.49684E-05	111731
0.3	0.257584472	2.09315E-05	154885
0.4	0.355735966	6.76109E-06	193073
0.5	0.338689685	8.554E-06	227410

TotPG (total phosphoglycerides)

thresh	pval	r2	nsnps
0.001	0.274423856	2.65834E-05	1410
0.05	0.011235683	0.000385229	34882
0.1	0.016801795	0.000333901	62721
0.2	0.008918162	0.000415045	111438
0.3	0.030079688	0.000261301	154460
0.4	0.033900182	0.000246695	192528
0.5	0.064711747	0.000170073	226766

BHBA (Ketone bodies)

thresh	pval	r2	nsnps
0.001	0.177291564	0.000116258	868
0.05	0.05452605	0.000348319	20369
0.1	0.076693928	0.000276482	36491
0.2	0.070632294	0.000293557	64866
0.3	0.046115741	0.00038454	89563
0.4	0.076851492	0.000276059	111587
0.5	0.060191964	0.000327218	131215

References

1. Sud, M. *et al.* LMSD: LIPID MAPS structure database. *Nucleic Acids Res.* **35**, D527-32 (2007).
